# Engineering Layered Magnetic Hydrogels for Cell Placement via Shear and Magnetic Field‐Induced Assembly

**DOI:** 10.1002/adma.73847

**Published:** 2026-07-01

**Authors:** Guillermo Camacho, Jose R. Morillas, Jesús García‐Gutiérrez, Stefania Nardecchia, Óscar Martínez‐Cano, Juan de Vicente

**Affiliations:** ^1^ F2N2Lab, Magnetic Soft Matter Group, Department of Applied Physics, Faculty of Sciences University of Granada Granada Spain

**Keywords:** anisotropic hydrogel, biomaterial, bioprinting, magnetic hydrogel, magnetorheological fluid, lamella, layer, pattern, tissue engineering

## Abstract

The design of hydrogel‐based artificial tissues capable of reversible, programmed, and complex motions requires both stimuli‐responsiveness and structural anisotropy. In this work, non‐unidirectional anisotropies are generated in biocompatible hydrogels by structuring magnetic particle suspensions into lamellar architectures through two distinct routes: the application of an unsteady magnetic field to a quiescent sample, and the superposition of a steady magnetic field with shear flow. In both approaches, magnetic particles undergo directed self‐assembly within a polymer matrix that subsequently gels, thereby preserving the formed structures. We analyze the assembly kinetics, characterize the resulting lamellar patterns, and construct phase diagrams for each method. The morphology and periodicity of the lamellae are shown to depend strongly on geometric confinement, enabling tunable interlamellar spacing from tens to hundreds of microns. Crucially, it is demonstrated that the resulting layered hydrogels can confine human fibroblasts between adjacent particle‐rich lamellae, maintain cell viability above 95% over 7 days of culture, and promote preferential cell alignment parallel to the layered structures. These findings establish magnetic field‐directed lamellar structuring as a versatile route to anisotropic hydrogels with programmable internal architecture, opening new opportunities in tissue engineering, bioactuation, and soft robotics.

## Graphical abstract

Magnetic particles are organized into layered architectures by combining shear flow and magnetic fields, with the resulting structures governed by appropriate Mason numbers. The programmed assemblies provide spatial guidance for cell placement, linking field‐controlled self‐assembly, flow‐induced structuring and biological organization. This strategy offers a versatile route to engineer anisotropic magnetic soft matter with designed microstructure and function.

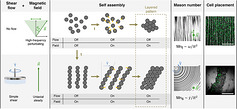

## 1 Introduction

Biological tissues are hierarchically organized, anisotropic composites, structured from the molecular to the macroscopic scale. This intrinsic anisotropy underlies many of their macroscopic properties, which are essential for the complex functions of living organisms [1, 2]. A clear example is skeletal muscle, which exhibits unidirectional anisotropy through its multiscale, oriented fiber architecture. This structure, essential for joint mobility, originates from the self‐assembly of collagen proteins into directionally aligned bundles. At the microscopic level, muscle contraction arises from the ordered arrangement of actin and myosin filaments in the sarcomere, while at the macroscopic scale, this organization imparts mechanical strength, enabling directional force transmission and shock absorption. Motor proteins contribute to this process by guiding the alignment of collagen bundles, generating controlled mechanical forces and promoting energy dissipation [3].

A further level of structural complexity in biological tissues involves bidirectional anisotropy, as observed in articular cartilage. Cartilage is characterized by a biphasic composition: a liquid phase rich in water and a solid matrix of collagen and proteoglycans. Its remarkable mechanical performance—high strength, low friction, and efficient shock absorption—derives from a multilayered collagen fiber network. The superficial layer, adjacent to the joint cavity, contains collagen fibers arranged parallel to the surface and orthogonal to each other, providing shear resistance. The intermediate layer features randomly oriented fibers, enhancing compressive strength, while the deep layer exhibits crosshatched fibers, optimizing load‐bearing capacity. This stratified anisotropy allows cartilage to simultaneously ensure joint stability and mobility [4].

Tissue engineering seeks to replicate such functional architectures in artificial constructs. Traditional approaches involve seeding cells into biomimetic three‐dimensional (3D) hydrogels that mimic the extracellular matrix and offer structural and biochemical cues for cell growth. However, most conventional hydrogels are isotropic and homogeneous, limiting their ability to emulate the directional and hierarchical organization of native tissues—particularly where aligned cellular architecture and long‐range order are critical [5]. Achieving spatiotemporal control over cellular organization within scaffolds remains a major challenge [6].

Cell behavior—proliferation, migration and differentiation—is profoundly influenced by the anisotropy of the surrounding matrix. Reproducing such anisotropy in engineered hydrogels is therefore a central goal. Prevalent strategies involve orienting polymer chains, creating unidirectional, channel‐like voids, or aligning nanofillers (not necessarily spherical in shape) within a precursor solution, followed by gelation to preserve the induced structure [7]. These architectures are typically induced by directional stimuli such as mechanical forces (shear, tension or compression) or vector fields (electric or magnetic) [8]. Among these, magnetic fields stand out due to their non‐contact, non‐invasive nature and their ability to uniformly penetrate materials, enabling the fabrication of macroscopically thick, aligned hydrogels. Once aligned, the structures are fixed through polymerization or cross‐linking. These anisotropic hydrogels have shown great promise in applications such as oriented cell culture [9] and directed mass transport [10].

More intricate anisotropic designs include multilayered and patterned hydrogels [8]. Multilayer structures are created by sequential casting and gelation of precursor solutions, resulting in distinct layers with tailored mechanical or chemical properties. Patterned structures, meanwhile, are fabricated via photolithography or 3D printing, enabling spatial control over fiber orientation at various scales. Both strategies allow the integration of non‐unidirectional anisotropies—layered, or spatially varying—within a single construct. Such architectures, typically larger than individual cells, have also been produced through time‐intensive polymerization steps under dynamically changing magnetic fields, allowing spatial tuning of macroscopic anisotropy [11].

Magnetic hydrogels are generally fabricated via three main strategies: blending, in situ precipitation, and grafting, with blending being the most widely used due to its simplicity and versatility [12]. Various techniques exist for introducing ordered microstructures, including magnetic‐field‐induced assembly, microfluidics, and 3D printing. Most efforts focus on inducing unidirectional anisotropy by aligning magnetic nanoparticles (spheres, rods, or platelets) into chain‐like assemblies under a magnetic field [5]. The morphology and orientation of these chains depend on factors such as particle size, concentration, and the strength, direction, and gradient of the applied field [12]. Recently, magnetic field frequency has emerged as a critical parameter for dynamically controlling anisotropy, enabling the formation of complex structures beyond simple chain‐like assemblies, such as spirals and foam‐like architectures [13, 14].

Despite progress, generating bidirectionally anisotropic hydrogels remains challenging, with only a few successful examples. Liu et al. [15] demonstrated a hydrogel with embedded titanate nanosheets that aligned cofacially under an electric field due to electrostatic repulsion. The resulting material displayed directional mechanical behavior; flexible under shear parallel to the nanosheets but resistant under orthogonal compression. In another study, Lee et al. [16] achieved layered magnetic architectures by suspending ferromagnetic microparticles in a rotating viscous polymer. Under a static magnetic field, the particles self‐assembled into periodic open‐lattice structures, fixed by thermal curing. This strategy enabled the formation of 3D architectures with tunable spacing, controlled by magnetic particle concentration. Both approaches illustrate innovative paths toward non‐unidirectional anisotropic materials with biomimetic functionality.

**FIGURE 1:**
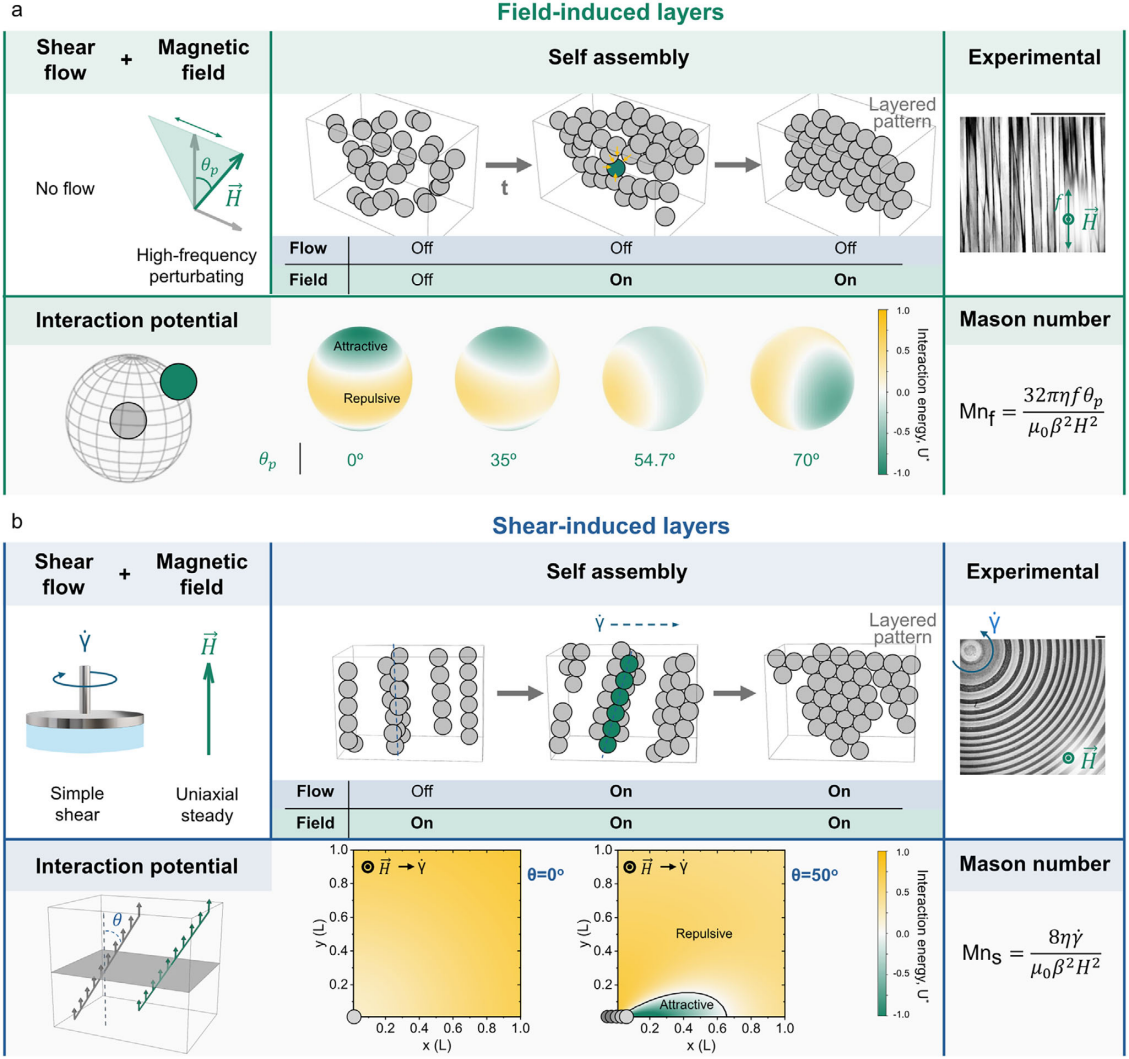
Comparative schematics of field‐induced and shear‐induced layered assembly. (a) Field‐induced layer assembly. A quiescent suspension is subjected to a high‐frequency perturbating magnetic field composed of a steady component and an orthogonal oscillatory component. At sufficiently high Mn_f_, particles cannot follow the instantaneous field vector and experience an effective time‐averaged interaction. The corresponding interaction potential shows in‐plane attraction and out‐of‐plane repulsion, promoting the formation of lamellar structures in the field plane. (b) Shear‐induced layer assembly. A suspension prestructured by a uniaxial steady magnetic field is subjected to shear flow with the field aligned along the velocity gradient. The flow tilts the field‐assembled chains, modifying the inter‐chain interaction potential and generating attraction within the flow‐gradient plane, which promotes layer formation. Representative top‐view micrographs of the final layered structures are shown on the right column. Black scale bar: 500 µm.

In this study, we explore layered pattern formation in magnetic particle suspensions under two configurations (Figure 1): i) a quiescent suspension subjected to a perturbating magnetic field, referred to here as field‐induced assembly, and ii) a suspension under steady shear flow and a uniaxial steady magnetic field aligned with the velocity gradient, referred to as shear‐induced assembly. In both cases, the key control parameter is the Mason number (Mn), a dimensionless quantity comparing hydrodynamic and magnetostatic effects, although the origin of the hydrodynamic forcing differs in the two configurations. The detailed definitions of the field‐induced and shear‐induced Mason numbers are given in Sections 2.1 and 2.2, respectively.

In the last section of this work, the resulting layered structures are immobilized by in situ gelation in a polymer matrix, effectively “freezing” the particle‐based architectures within a biocompatible hydrogel. Two distinct polymer matrices were employed, each tailored to the specific requirements of the corresponding assembly route. For the field‐induced approach, a composite hydrogel based on oxidized laminarin and gelatin was used. Gelation in this system proceeds through dynamic covalent imine (Schiff‐base) bond formation between the aldehyde groups of oxidized laminarin and the amino groups of gelatin, enabling rapid and cytocompatible crosslinking under physiological conditions without external triggers. For the shear‐induced approach, a physical gelatin hydrogel was employed, in which solidification is induced by lowering the temperature below the gelation threshold, thereby promoting physical crosslinking of the polymer chains. In both cases, the matrix undergoes the sol‐to‐gel transition while the assembled particle structures are maintained, preserving the lamellar architecture generated during structuration. We then demonstrate the utility of these patterned scaffolds for tissue‐engineering applications by introducing human fibroblast cells and confining them within the interlamellar regions.

Although both routes lead to lamellar architectures, the physical origin of the attractive interactions is fundamentally different: in the field‐induced case, it arises from a time‐averaged dipolar interaction under a rapidly varying field, whereas in the shear‐induced case it results from flow‐driven tilting of field‐assembled chains. This mechanistic contrast is summarized in Figure 1 and developed comparatively throughout Sections 2.1 and 2.2.

## 2 Results and Discussion

**FIGURE 2:**
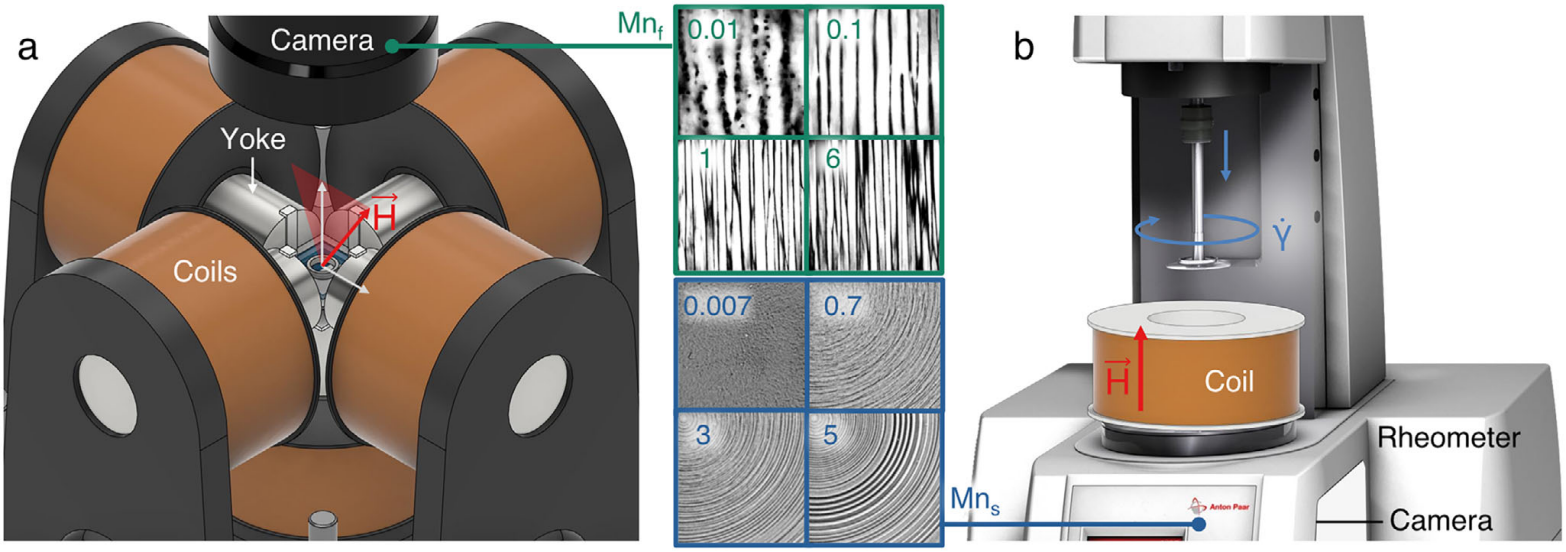
Experimental configurations and representative structures formed at different Mason numbers. (a) Field‐induced assembly: A perturbating magnetic field is applied using a custom‐built triaxial magnetic field generator capable of producing high‐frequency fields along all three spatial axes. Top‐view images are acquired with a high‐speed camera. Top snapshots on the right show representative microstructures at different values of Mn_f_. (b) Shear‐induced assembly: A steady shear flow is imposed using a commercial torsional rheometer with a plate‐plate geometry, while a uniaxial steady magnetic field is applied via a surrounding coil aligned with the velocity gradient direction. Micrographs are captured from below through the transparent bottom plate using a high‐speed camera. Bottom snapshots on the left show representative microstructures at different values of Mn_s_.

Figure 2 shows the experimental setups used in the generation of layered structures and representative assemblies formed at different values of Mn in a dispersion of magnetic particles in a Newtonian liquid carrier (Experimental Section). At low Mn, particles arrange into chain‐like structures. However, as Mn increases beyond a threshold, well‐defined periodic layered patterns emerge. These patterns develop either in the plane of the perturbating magnetic field (field‐induced case) or in the plane defined by the velocity field and its gradient (shear‐induced case). Remarkably, despite the different symmetries of the driving fields, the resulting structures exhibit notable similarities. To gain insight into the formation and evolution of these patterns, we analyze the underlying mechanisms and dynamics in both configurations in the following subsections.

### 2.1 Field‐Induced Assembly: Quiescent Suspensions Subjected to a Perturbating Magnetic Field

Perturbating magnetic fields consist of the superposition of a uniaxial steady magnetic field ${\vec{H}}_{\mathrm{st}}$ and an orthogonal oscillatory field ${\vec{H}}_{\mathrm{os}}$ (Figure 2a). This specific field configuration was likely first explored by Donado and coworkers [17], who demonstrated that a small‐amplitude, low frequency, orthogonal sinusoidal field promotes lateral interactions among chain‐like aggregates, thereby enhancing their thickness and rigidity and resulting in improved rheological performance. For sufficiently high particle concentrations, perturbation amplitudes, and frequencies, particles assemble into planar layers aligned with the field plane [18, 19], exhibiting markedly enhanced rheological properties [22]. However, a detailed and systematic investigation of the effects of high‐frequency perturbating fields on structure formation and dynamics remains lacking in the literature.

The field‐induced Mason number, which quantifies the ratio of hydrodynamic to magnetostatic forces in this configuration, is defined as $Mn_{f}=\frac{32\pi \eta f\theta_{p}}{\mu_{0}\beta^{2}H^{2}}\;$[17, 18, 19]. Here, η is the viscosity of the carrier fluid, *f* the frequency of the perturbating field, and θ_*p*_ the perturbation angle, that is, the maximum angle swept by the field. β is the magnetic contrast factor of the particles β  = (μ_*p*_ − μ_0_)/(μ_*p*_ + 2μ_0_) , μ_*p*_ (μ_0_) is the magnetic permeability of the particles (carrier), and *H* the external field strength averaged over a field cycle. The specific values of the magnetic field strength, frequency, perturbation angle, and corresponding Mn_f_ used in the different field‐induced experiments are summarized in Table 1 in the Experimental section.

**TABLE 1:** Experimental parameters and Mason numbers for field‐induced and shear‐induced assembly. Specific experimental parameters used in field and shear‐induced assembly experiments and corresponding field and shear‐induced Mason number Mn_f_ and Mn_s_, respectively.

	Experiment	Mn_*f*_	θ_*p*_ (°)	*H* (kA·m^−1^)	*f* (Hz)	η (mPa·s)
Field‐induced	Phase diagram	0.01–6	10–90	8	0.3–1700	7
	Layered hydrogels	3.6–5	54.7–70	12.3	100	30 (initial)
	**Experiment**	**Mn**_***s***_		***H*** **(kA·m^−1^)**	$\overset{.}{\gamma }$ **(s^−1^)**	**η** **(mPa·s)**
Shear‐induced	Phase diagram	0.0001‐60		5.4–16.3	0.01‐1000	106
	Layered hydrogel	3.3		10	200	100 (initial)

Next, we present the governing interparticle interactions, detail the assembly dynamics under perturbating fields, and construct a phase diagram of the observed structures as a function of θ_*p*_ and Mn_f_.

#### 2.1.1 Potential Energy of Interaction Under Perturbating Fields

In the presence of a uniaxial steady magnetic field, magnetizable particles interact via the classical magnetostatic dipole‐dipole potential. In the linear regime, this interaction is highly anisotropic and favors the formation of chains aligned with the direction of the magnetic field [23]:

(1) $$U^{dip}\left(\vec{r}\right)=\frac{\mu_{0}\pi d^{6}\beta^{2}H_{st}^{2}}{16\;r^{3}}\;\left(1-3\cos^{2}\theta \right)$$

where *d* is the particle diameter, $r=|\vec{r}|\;$ is the center‐to‐center distance between two particles, and θ is the polar angle between $\vec{r}$ and ${\vec{H}}_{\mathrm{st}}$.

We now consider a superimposed oscillatory field in the orthogonal direction, $\vec{H}\;\left(t\right)=H_{st}\;\hat{z}+H_{os}\sin\omega t\;\hat{x}$. For low forcing frequencies, particles can follow the instantaneous field direction, but for sufficiently large Mason number Mn_f_, the field varies too rapidly and the particles experience an effective time‐averaged interaction instead. In the linear response regime, the dipolar pair potential averaged over one field cycle becomes (Note S1):

(2) $$\langle U^{dip}\left(\vec{r}\right)\rangle =\frac{\mu_{0}\pi d^{6}\beta^{2}H_{st}^{2}}{16\;r^{3}}\;\left[1-3\cos^{2}\theta +\frac{\tan^{2}\theta_{p}}{2}\left(1-3\sin^{2}\theta \cos^{2}\varphi \right)\right]$$

where φ is the azimuthal angle of $\vec{r}$ in the plane of the oscillatory field, and θ_*p*_ is defined by tan θ_*p*_ = *H_os_*/*H_st_* , representing the maximum angle swept by the net field. The first two terms correspond to the classical dipolar interaction from the steady component, while the third term, proportional to tan^2^θ_*p*_, arises from the oscillatory component and introduces attractive in‐plane interactions for appropriate orientations.

These in‐plane attractions and out‐of‐plane repulsions promote the formation of layered structures, particularly when magnetic interactions dominate over thermal agitation (i.e., for large Lambda ratios λ; see [24]), and the particle area fraction $\phi_{2D}$ is sufficient for dense aggregation [25], akin to behaviors observed in rotating fields [26]. Notably, at the magic angle, when θ_*p*_ = θ_*m*_  = 54.7°, the interaction becomes completely isotropic in the field plane, equivalent to a rotating field (see Figure S1.1, Note S1). Under such conditions, the radial pair potential can be extracted experimentally using the inverted Boltzmann distribution [27]:

(3) $$\frac{U\left(r\right)-U\left(r_{ref}\right)}{k_{B}T}=\ln\left[\frac{n\left(r_{ref}\right)}{n\left(r\right)}\right]\;$$

Here, *n*(*r*) is the interparticle distance probability distribution, which is normalized to a reference point *r_ref_*, *k_B_* is the Boltzmann constant and *T* the temperature of the system. As shown in Figure S1.2 in the Note S1, the pair interaction obtained at θ_*p*_ = θ_*m*_ matches well with Equation 3. While multibody effects due to mutual polarization may slightly alter the interaction landscape, the pair potential adequately predicts the emergence of layered structures [28].

#### 2.1.2 Self‐Assembly and Dynamic Scaling Theory

The aggregation dynamics of magnetic suspensions—both Brownian and non‐Brownian—under steady [29, 30] and rotating [31] fields have been shown to follow the Dynamic Scaling Theory (DST) proposed by Vicsek and Family [32], wherein clusters grow hierarchically following a power‐law. Recent work by Ben Salah et al. [25] confirmed that, for fixed θ_*p*_ = 45°, aggregation within layers proceeds via wall‐edge coagulation and conforms to DST. However, the influence of the perturbation angle θ_*p*_ on the self‐assembly pathway remains unexplored.

In this study, the clustering dynamics under high‐frequency perturbating fields were investigated experimentally and via particle‐level simulations (Note S2). The evolution of the mean cluster size *S*(*t*) and the total number of clusters *N_c_* (*t*) was tracked, where:

(4) $$S\left(t\right)=\frac{\sum s^{2}n_{s}}{\sum s^{\circ }n_{s}}\;,N_{c}\left(t\right)=\sum n_{s}$$

and *n_s_* denotes the number of clusters containing *s* particles. According to DST, the cluster size distribution follows:

(5) $$n_{s}∼s^{-2}f\left(\frac{s}{t^{z}}\right)$$

where *f*(*x*) ∼ *x* ^Δ^ for *x* ≪ 1, with *z* and Δ being the kinetic and crossover exponents, respectively. This implies power‐law scaling of the clustering parameters: *S*(*t*) ∼ *t^z^* and *N_c_* (*t*) ∼ *t* ^−*z*′^. In diffusion‐dominated systems, *z* is typically constant: *z* ≈ 1.4 for isotropic diffusion and *z* ≈ 0.5 for anisotropic cases [33]. In magnetically dominated systems, however, *z* can depend on λ and ϕ.

**FIGURE 3:**
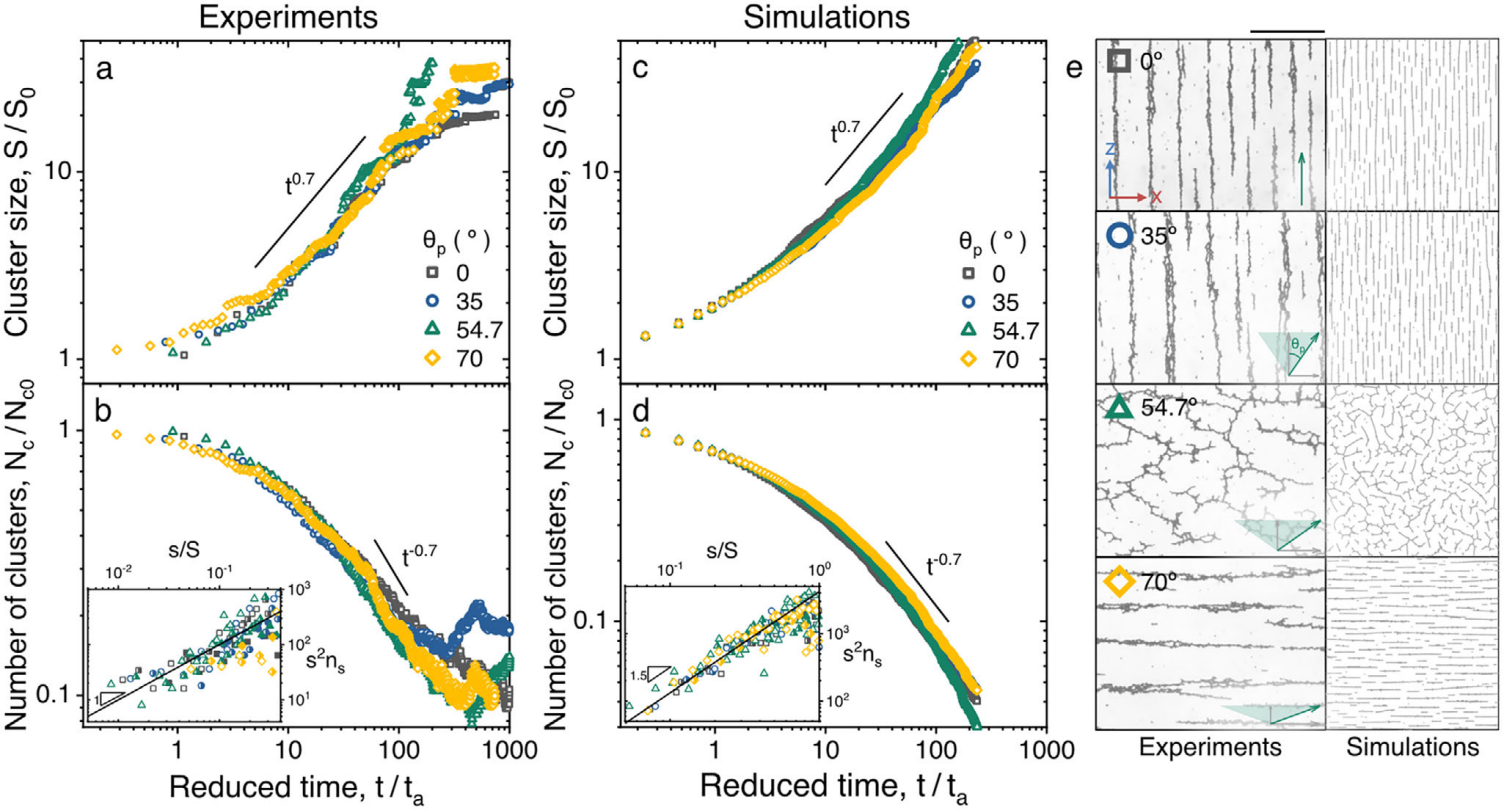
Clustering dynamics and dynamic scaling under perturbating magnetic fields for different perturbation angles θ_*p*_. Average cluster size *S*(*t*) and number of clusters *N_c_* (*t*) for the experiments (a,b) and particle‐level simulations (c,d), respectively. Data are normalized to their initial value and the magnetic aggregation timescale *t_a_*. The insets include the scaling functions *f*(*s*/*S*) ∼  *s* ^2^ *n_s_* that show a power law behavior for *s*/*S* ≪ 1. Black lines include power law scaling for visual guidance. (e) Micrographs (left column) and simulation snapshots (right column) of the assembled clusters at *t*/*t_a_* = 250. Black scale bar represents 100 µm. Magnetic field strength was set to 2 kA ·m^−1^ with a frequency of *f*  = 100 Hz and a surface particle loading of $\phi_{2D}$ = 0.1.

Figure 3 presents the time evolution of clustering parameters at $\phi_{2D}$ 0.1 for various θ_*p*_, under a high‐frequency field in the plane of the suspension (Mn_f_ ∼ 10). Both experimental and simulation results are normalized by the magnetic aggregation timescale *t_a_* [34], defined as:

(6) $$t_{a}=\frac{2\eta }{5\mu_{0}\beta^{2}H^{2}}\;{\left(\frac{\pi }{6\phi_{2D}}\right)}^{\frac{5}{2}}$$

The absence of significant pre‐existing agglomeration is consistent with the unreduced clustering data, which start from an average cluster size close to one particle before field‐induced aggregation begins.

To assess the robustness of this scaling, additional experiments were conducted by varying $\phi_{2D}$. The resulting changes in the power‐law exponents were negligible (Note S3), suggesting that, within this high‐frequency regime, the surface fraction has only a minor effect on the aggregation kinetics.

As shown in Figure 3a–d, the clustering dynamics collapse onto a master curve regardless of θ_*p*_, confirming angle‐independence of the early‐stage assembly kinetics. Slight deviations arise at longer times, particularly for θ_*p*_ = θ_*m*_, where isotropic interactions enable percolation and larger aggregate growth (see Figure 3e). The extracted kinetic exponent *z* ∼ 0.7 agrees well with previous studies under steady [30] and rotating fields [31, 35].

Insets in Figure 3a–d show the scaling functions *f*(*s*/*S*) ∼  *s* ^2^ *n_s_*, confirming the power‐law behavior predicted by DST, with Δ ∼ 1 in experiments and Δ ∼ 1.5 in simulations. Micrographs and simulation snapshots (Figure 3e) reveal how the anisotropy of magnetic interactions, governed by θ_*p*_ (see Equation 2), controls aggregate morphology. At low θ_*p*_, interactions are strongly anisotropic, akin to those under uniaxial steady fields. At θ_*p*_ = θ_*m*_ , interactions become isotropic, producing isotropic networks at low concentrations and compact, disk‐like clusters at higher particle loadings. At even higher θ_*p*_, anisotropy re‐emerges but is now aligned with the perturbation direction. These observations suggest that fine‐tuning in‐plane anisotropy could enable layer formation at higher volume fractions in 3D systems [36].

#### 2.1.3 Phase Diagram

**FIGURE 4:**
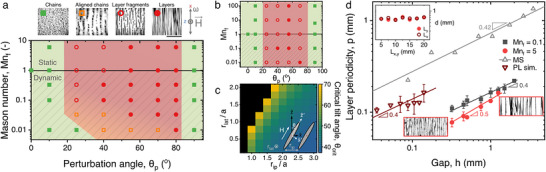
Phase behavior and confinement‐dependent periodicity in field‐induced assembly. (a) Experimental phase diagram of the suspensions under perturbating magnetic fields as a function of perturbation angle θ_*p*_ and field‐induced Mason number Mn_f_, showing three principal structural regimes: chains along the dominant field direction (for θ_*p*_ ∼  0° and 90°), layered phases (fragmented and percolating) at intermediate angles, and aligned chains at ultra‐low Mn_f_. (b) Corresponding phase diagram from particle‐level simulations. (c) Map of the critical tilt angle θ_*crit*_ for the aligned chains model, showing the minimum angle required for attractive interaction as a function of the in‐plane (*r_ip_*) and orthogonal (*r_lat_*) distances between two ellipsoids. The inset illustrates the geometry of the ellipsoid model. (d) Layer periodicity as a function of sample height *h*. Magnetostatic calculations (MS) and particle‐level simulations (PL; both in open symbols) agree with experimental data (solid symbols) obtained under layer‐forming conditions (θ_*p*_ = 54.7°). The inset demonstrates a negligible influence of sample dimensions perpendicular to gravity. Field strength *H*  = 8 kA m^−^^1^ and particle volume fraction ϕ= 0.01.

After sufficiently long times, the suspension reaches a steady–state configuration, from which the final assembled structures are evaluated. To systematically identify the conditions for layer formation, we constructed a phase diagram as a function of θ_*p*_ and Mn_f_ (see Table 1 in Experimental section for specific field conditions and corresponding Mn_f_ values). Figure 4a presents the experimental phase diagram at a fixed particle volume fraction ϕ=0.01. This concentration was chosen to ensure structural percolation under the conditions employed—namely, a sample cell height of 1 mm and a magnetic field strength of 8 kA·m^−^^1^—consistent with an earlier work [37]. Importantly, increasing the particle concentration does not lead to qualitatively different structural phases; rather, it enhances the connectivity of the system, promoting the transition from fragmented to fully percolating layers due to stronger interparticle interactions. This behavior is further corroborated by particle‐level simulations, which exhibit excellent agreement with the experimental phase boundaries (see Figure 4b), reinforcing the reliability and generality of the observed assembly regimes.

Depending on the perturbation angle θ_*p*_, two primary assembly regimes are identified, involving chain or layer formation. Parallel chains (low θ_*p*_): At small angles, the perturbation field is too weak to overcome the lateral repulsion induced by the steady field. As a result, particles form linear chains aligned with the steady field direction, as typically observed under uniaxial steady fields [38]. Layered structures (intermediate $\theta_{p}\;∼$ 25°‐80°): Here, the perturbation component generates in‐plane attractive interactions, promoting the assembly of parallel layers. Depending on the particle concentration, these layers may appear as fragmented domains (at low concentrations) or be fully percolating (at high concentrations). In the transitional region near regime boundaries, non‐percolating layer fragments extend along the field plane, exhibiting strong anisotropy inherited from the field configuration. Orthogonal chains (high θ_*p*_): At large perturbation angles, the oscillatory component dominates over the steady one, causing chains to realign with the time‐varying field. In this regime, the absence of a steady field in the gravity direction leads to rapid sedimentation due to the high density mismatch between iron particles and the carrier fluid, which also hinders optical measurements through the sample because of negligible light transmission.

The Mason number Mn_f_ plays a critical role in dictating the dynamic character of the structures. At low Mn_f_ (*f* ≲ 100 Hz), structures exhibit dynamic oscillations in response to the field (Movie S1). As Mn_f_ increases, these oscillations become progressively damped due to viscous stresses, in line with observations in other unsteady field configurations [36, 39]. At high Mn_f_ (*f* ≲ 100 Hz), viscous drag prevents particles from tracking the instantaneous field vector, resulting in quasi‐static, time‐averaged interactions that stabilize static assemblies.

Remarkably, at extremely low Mn_f_  ∼ 0.01, a distinct phase emerges, which we term “aligned chains” phase. In this regime, particles form thick chains that tilt synchronously with the instantaneous magnetic field vector, yet remain arranged in parallel planes without merging into continuous layers. To rationalize this behavior, we model these chains as magnetized ellipsoids and estimate their pairwise interaction energy. Assuming collinearity between the ellipsoid's long axis, the local magnetization and the magnetic field, the magnetic potential generated by an ellipsoid can be expressed as:

(7) $$V=-\frac{3}{8\pi }\frac{mV_{0}}{H}\int_{\xi }^{\infty }\frac{ds}{\left(s+a^{2}\right)\sqrt{\left(s+a^{2}\right){\left(s+b^{2}\right)}^{2}}}$$

in ellipsoidal coordinates (ξ,  η,  ζ) [40, 41], where *a* and *b* are the semi‐major and semi‐minor axes, respectively, *m* is the ellipsoid magnetization, and *V*_0_ is the external field potential. The resulting magnetic field ${\vec{H}}_{\mathrm{ell}}=-\nabla V$ is then used to compute the interaction energy between two oscillating ellipsoids as $U_{\mathrm{ac}}=-\mu_{0}\vec{m}\cdot {\vec{H}}_{\mathrm{ell}}$. The analysis reveals that when the ellipsoids are vertically aligned with the field (i.e., ω*t* ∼ *n*π), the interaction is repulsive (as the case of uniaxial steady fields). Conversely, when tilted (e.g., $\omega t∼(n+\frac{1}{2})\pi$), the interaction remains repulsive at short distances but can become attractive at long distances around the field plane. This long‐distance attraction is conditional, appearing only when both the inter‐ellipsoid distance and tilting (i.e., perturbation) angle exceed critical thresholds. A phase map of this threshold tilt angle θ_*crit*_ for attractive interactions is represented in Figure 4c as a function of in‐plane (*r_ip_*, in the field plane) and out‐of‐plane (*r_lat_*, perpendicular to the field plane) separation. Interaction never becomes attractive at short distances, and, at long distances, the attraction threshold is significantly lower for ellipsoids aligned with the field plane than for those that are not. This explains the formation of aligned yet non‐merging thick chains in this regime. Note S4 provides a detailed analysis of these critical conditions, including detailed derivations and maps of the interaction potential showing the attractive and repulsive regions for different tilting angles.

#### 2.1.4 Period of the Layered Pattern

At high Mn_f_, the layers formed within the suspension exhibit a well‐defined periodic spacing. This structural periodicity is of particular interest for applications requiring bidirectional anisotropy, such as in tissue engineering, where the ability to tune the spacing between layers is critical for confining and guiding biological elements [6].

We found that the period of the layered structures depends primarily on the degree of confinement, increasing with the vertical gap height *h*, consistent with observations in uniaxial steady fields [37]. Figure 4d shows the measured layer spacing as a function of the sample chamber height for perturbation angle θ_*p*_ = 54.7°, under two different values of Mn_f_. A clear power‐law dependence emerges, with the layer period scaling as ≈ *h* ^0.4^. However, for large confinement gaps (*h* > 2 mm), the system undergoes depercolation at the employed particle concentration, leading to anomalously increased layer spacing (data not shown in Figure 4d).

This power‐law behavior is well captured by magnetostatic calculations of dipole arrays, also presented in Figure 4d as open triangles. In these calculations, preassembled layers of varying thickness were considered, and the system's equilibrium configuration was determined by minimizing its total magnetic energy (Note S5). Particle‐level simulations further corroborate these findings (see Figure 4d), yielding a similar scaling exponent and supporting the robustness of the observed trend. It is worth noting that the lateral dimensions of the sample chamber (i.e., in the direction orthogonal to the steady field component) do not significantly influence the layer spacing, as demonstrated by the negligible variation shown in the inset of Figure 4d. This sublinear confinement dependence contrasts with the approximately linear scaling observed for shear‐induced layers (Section 2.2), underscoring that similar lamellar morphologies can emerge from distinct interaction mechanisms and stabilization pathways.

### 2.2 Shear‐Induced Assembly: Steady Shear Flow Combined with a Uniaxial Steady Magnetic Field Aligned with the Velocity Gradient

Having examined the formation of magnetic layered structures under perturbating fields, we now address the case of structure formation under the superposition of steady shear flow and a uniaxial steady magnetic field. In the configuration explored in this work, the magnetic field is applied in the same direction as the velocity gradient of the shear flow (see Figure 2b). While the emergence of layered structures in this geometry has been previously reported [42], notable discrepancies remain between theoretical predictions and experimental observations [43, 44]. Furthermore, to the best of our knowledge, no systematic comparisons have been made between these structures and those assembled under perturbating magnetic fields.

In this configuration, the shear‐induced Mason number quantifying the ratio of hydrodynamic to magnetostatic forces is defined as $Mn_{s}=\frac{8\eta \dot{\gamma }}{\mu_{0}\beta^{2}H^{2}}\;$[20, 21], being $\dot{\gamma }$ the imposed shear rate.

In the following subsections, we describe the governing inter‐chain interactions, analyze the dynamic assembly process under shear, and construct a phase diagram of the resulting structures as a function of the shear rate and Mn_s_.

#### 2.2.1 Potential Energy of Interaction Under Shear‐Field Superposition

Under a steady magnetic field, particles assemble into chains following the anisotropic dipolar interaction of Equation 1. In the combined shear‐field configuration, a recent theoretical model [44] predicts the formation of layered structures originating from preassembled chains aligned by the magnetic field. As Mn_s_ increases, the applied shear flow progressively tilts these chains toward the flow direction. This tilt alters the nature of interchain interactions; lateral repulsion between chains is reduced and can become attractive, ultimately driving their aggregation into extended layers aligned with the flow. Notably, this mechanism contrasts with that observed under perturbating magnetic fields, where the tilting of chains is induced by the field itself. Here, in a fundamental shift of control, it is the balance between magnetic field and shear flow that governs the orientation and subsequent assembly of the chains.

**FIGURE 5:**
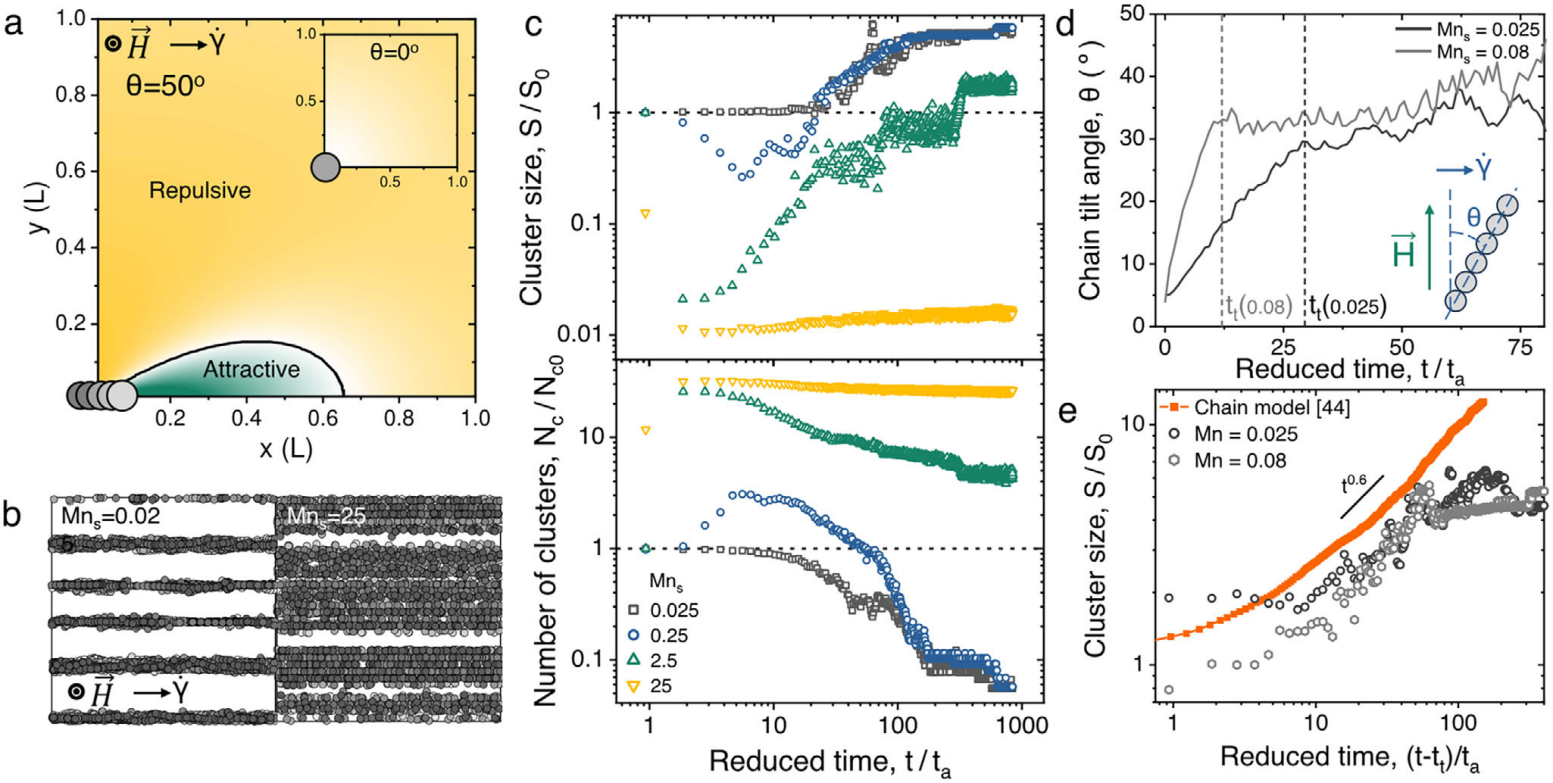
Interchain interactions, tilting dynamics, and shear‐induced layer formation. (a) Color map of the interaction potential in the flow‐vorticity plane between two chains tilted by θ  =  50° with respect to the magnetic field. Yellow regions correspond to repulsive interactions, while green regions indicate attraction. The boundary between the two regimes is marked by a solid line. Inset: interaction potential for θ  = 0° (chains aligned with the field), showing fully repulsive behavior. Potential is normalized to the distance decay for easier visualization $U^{*}=\frac{4\pi Lr^{3}}{\mu_{0}m^{2}}\;U$. (b) Simulation snapshots of magnetic suspensions under shear at various Mn_s_ values. Compact, thin layers are observed at low Mn_s_, whereas high Mn_s_ values yield thick, disordered, liquid‐like layers. (c) Temporal evolution of the mean cluster size *S*(*t*) (top) and number of clusters *N_c_* (*t*) (bottom). Time is normalized by the characteristic aggregation timescale *t_a_*. Volume fraction: ϕ= 0.1. Flow onset corresponds to *t*  = 0, applied to preassembled chains under uniaxial steady field. (d) Average tilting angle of the clusters, computed from the gyration tensor. Vertical dashed lines indicate the time point where the chains reach their steady inclination *t_t_*. (e) Comparison of cluster growth dynamics for the chain model and particle‐level simulations at low Mn_s_ regime. To compare both models, *t_t_* is considered as the initial point for particle‐level simulations as chain model assumes already tilted chains.

This modified interaction potential can be derived by approximating the sheared chains as lines of dipoles tilted by an angle θ with respect to the magnetic field. Assuming the magnetic field is along the *z*‐axis and the flow is along *x*‐axis, the interaction energy between two chains—in the chain reference system $\vec{r}\prime$—becomes:

(8)

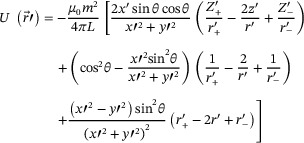

where *Z*′_±_ =  *z*′ ± 1, 
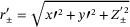
, with all distances scaled by the length of the chain *L*. See Note S6 for additional details on the expression and reference system. The resulting interaction force, shown in Figure 5a for a tilt angle θ  = 50°, exhibits a distinct attractive lobe in the flow direction. This supports the formation of layered structures within the flow‐field (velocity‐gradient) plane. In contrast, at θ  = 0° (no flow), the interaction remains entirely repulsive (see inset in Figure 5a).

#### 2.2.2 Self‐Assembly and Dynamic Scaling Theory

The directed self‐assembly of particles under uniaxial steady magnetic fields has been widely reported [30, 38, 45]. Several studies have also addressed the impact of superimposed shear flow on the resulting microstructures [46, 47, 48]. While simulations consistently predict the emergence of layers in both magnetorheological (MR) and electrorheological (ER) fluids under shear, significant quantitative discrepancies remain between simulations and experiments. In particular, layer formation in simulations typically occurs at Mn_s_ values orders of magnitude lower than those observed experimentally. Consequently, most simulation studies have focused on low shear regimes (Mn_s_ ≪ 1), whereas experimental observations of layering typically appear around Mn_s_ ≳ 1.

In this subsection, we analyze the layer formation dynamics through particle‐level simulations, as direct experimental tracking of individual particles or chains in dense suspensions under strong shear remains technically unfeasible because the suspensions are highly opaque under the particle loadings required for layer formation. The simulation protocol begins with the application of a uniaxial steady magnetic field, allowing chain formation. Subsequently, shear flow is imposed to promote layer development. Figure 5b shows representative simulation snapshots at various Mn_s_ values, while Figure 5c presents the temporal evolution of the mean cluster size, *S*(*t*), and the number of clusters, *N_c_* (*t*). Both metrics are referenced to the moment when shear is initiated (*t*  =  0), corresponding to the preassembled chain configuration.

Several clearly distinct assembly regimes emerge as a function of Mn_s_. Low shear regime (Mn_s_ = 0.025): The imposed shear does not disrupt the chains but instead causes them to rotate. This rotational motion enhances interchain attraction, leading to the gradual merging of chains into thin layers, typically one to two particles wide. In this regime, *S*(*t*) remains constant initially (rotation without merging), then increases rapidly as chains merge, eventually reaching a plateau indicative of a fully percolated layered structure. Intermediate shear regime (Mn_s_ = 0.25 and 2.5): The shear partially breaks the chains into shorter segments, resulting in an initial drop in *S*(*t*). However, these fragments rapidly reorganize into layers, and *S*(*t*) recovers as the structures coarsen. High shear regime (Mn_s_ = 25): Shear forces dominate over magnetic interactions, causing chain breakup and complete particle separation. This is reflected in a sharp decline in *S*(*t*). A subsequent slow reorganization process forms thick, disordered layers with fluid‐like internal structures. These “liquid layers” consist of loosely bound particles that do not significantly increase *S*(*t*), as dynamic bonds are continually broken by flow and reformed by the magnetic field. Movie S2 illustrates this dynamic behavior at Mn_s_ = 0.025 and 25.

A liquid phase at Mn_s_ > 1 was previously predicted by Melrose and Heyes [49], one of the few studies to explore this regime. However, they did not report the formation of any long‐range order, likely due to limitations in particle number (*N*  =  108–256) and simulation time. Our simulations reveal thick layered structures at high Mn_s_, which closely resemble those observed experimentally and fall within the expected Mn_s_ range.

In the low Mn_s_ regime, the observed behavior closely resembles the chain model described previously [44], where chains tilt under shear flow and subsequently merge into thin layers. This similarity allows for a meaningful comparison between the two systems, though several considerations must be addressed. The chain model assumes pre‐tilted chains as its initial condition. To compare the dynamics between both systems accurately, we must first quantify how chains tilt in particle‐level simulations. We computed the orientation of chains by measuring the angle between the field axis (*z*) and the main axis of the particle cluster, determined using the gyration tensor method [50]. Figure 5d shows this angular evolution, revealing that chains rapidly tilt upon shear application until reaching a steady orientation before merging begins. This steady angle agrees with the critical value of 39^°^ predicted by theoretical models [51], and also with the ranges observed experimentally [52, 53]. Although a direct in situ measurement of individual chain tilting under the present experimental conditions is not feasible because of the opacity of the concentrated suspensions under strong shear, the simulated steady inclinations remain consistent with the experimentally inferred tilt ranges reported for related field‐structured systems. This tilting process occurs more rapidly at higher Mn_s_ (in the low Mn_s_ regime, when no chain break is seen). We define the point where chains achieve their steady angle as *t_t_* (vertical lines in Figure 5d), and using this time as a reference point enables direct comparison between the chain model and particle‐level simulations. Cluster size evolution *S*(*t*) for both approaches is presented Figure 5e, demonstrating similar power‐law scaling of ≈ *t* ^0.6^ during chain merging. Particle‐level simulations saturate at significantly lower cluster size because the simulation domain size is limited due to the computational cost of considering each particle individually.

#### 2.2.3 Equilibrium Structures and Phase Diagram

**FIGURE 6:**
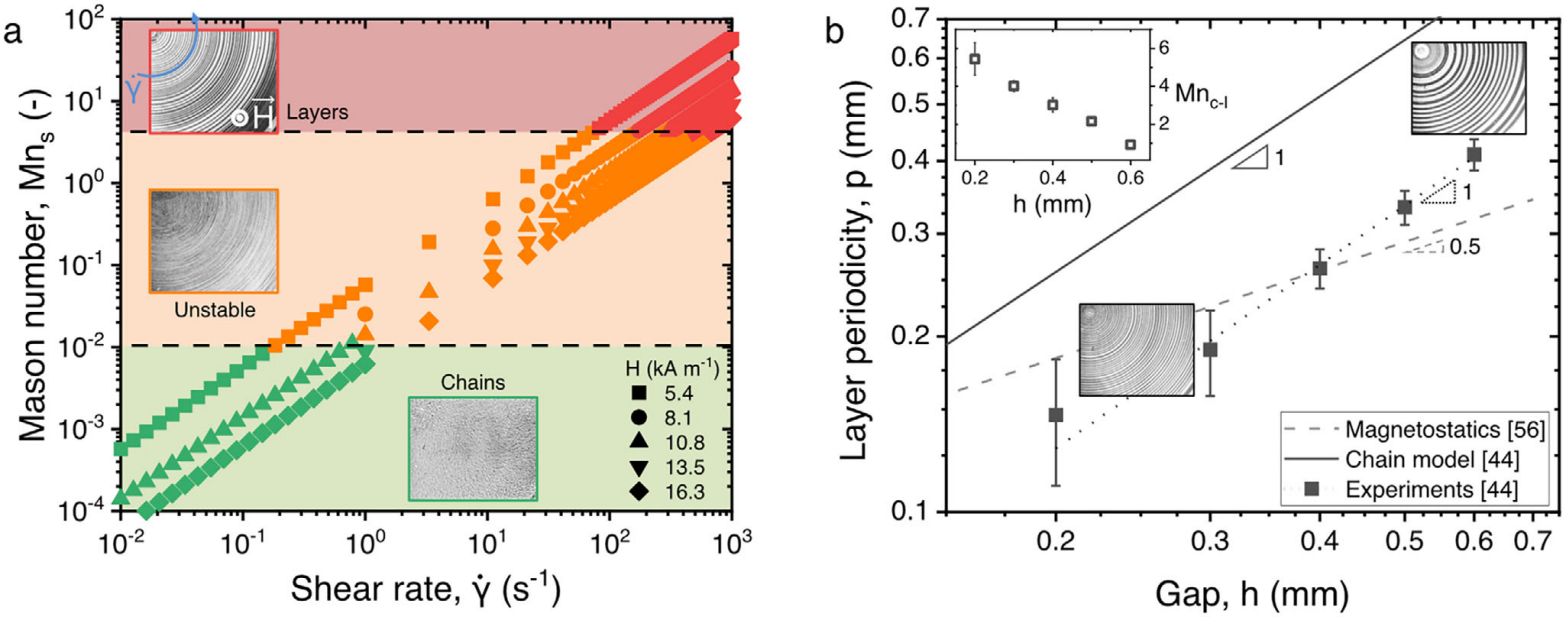
Structural phase diagram and confinement effects in shear‐induced assembly. (a) Structural phase diagram of the suspension under combined shear flow and magnetic field, as a function of Mn_s_ and shear rate $\dot{\gamma }$. Three distinct regimes are identified solely as a function of Mn_s_ for a given sample gap: field‐aligned chains for Mn_s_ < 0.01, unstable thin layers at intermediate Mn_s_ (0.01 < Mn_s_ < 4) that continuously form and break, and thick, stable layers aligned in the velocity–gradient plane for Mn_s_ ≳ 4. The horizontal dashed lines are included as guides to the eye. (b) Influence of sample confinement on the layered structures: periodicity of the pattern as a function of the gap height *h*. Experimental data (filled symbols) are compared with predictions from the magnetostatic model [Cutillas et al. [56], dashed line] and the chain‐tilting model [Morillas et al. [44], solid line]. The inset shows the critical Mason number for stable layer formation, Mn_c‐l_, as a function of gap height, revealing a negative linear relationship. For the gap used in this work (*h*  = 300 µm), the transition to stable layers occurs at Mn_c‐l_ ∼ 4. All experiments were performed at a particle volume fraction of ϕ= 0.1.

Stable layered structures under shear flow are experimentally observed for Mason numbers on the order of Mn_s_ ≳ 1. Importantly, startup tests have confirmed that these structures are not transient in nature [43]. At lower Mn_s_ values, the system exhibits thin, unstable layers that form and break apart continuously. A phase diagram summarizing these regimes is presented in Figure 6a, as a function of the Mason number Mn_s_ and shear rate $\dot{\gamma }$, derived from rheological measurements performed at varying magnetic field strengths.

Three distinct regimes are identified as a function of Mn_s_ alone for a given sample gap, which agree with those predicted by simulations. For low values (Mn_s_ < 0.01), particles remain in chains aligned with the magnetic field, which are not disrupted by the flow. At intermediate Mn_s_ (0.01< Mn_s_ < 4), unstable thin layers form and break intermittently. Only beyond Mn_s_ ≈ 4 does a stable pattern emerge that persists even as $\dot{\gamma }$ increases further. These stable layers exhibit a loose, fluid‐like internal structure, evidenced by their high surface coverage (≈ 40%), incompatible with a tightly packed configuration at the employed particle volume fraction (ϕ= 0.1).

It is important to emphasize that these Mn_s_ boundaries are only valid under conditions where magnetic and hydrodynamic interactions dominate the system behavior. That is, the system must be in a regime where Mn is the key control parameter. For this to hold, forces such as Brownian motion (λ > 1, Pe > 1) or gravity (*G* < 1) must be negligible compared to magnetic and shear‐induced forces [54, 55]. In our experiments, the applied magnetic field is sufficiently strong to meet these conditions.

#### 2.2.4 Period of the Layered Pattern

Beyond its dependence on Mn_s_, the formation of stable layered structures is strongly influenced by the sample gap height *h*. As shown in Figure 6b, the critical Mason number for layer formation Mn_c‐l_ decreases with increasing *h*, indicating that the true governing parameter for the onset of layering is not Mn_s_ alone, but rather the chain tilting angle, which scales as tan θ ∼ Mn_s_ · *h* ^2^ [44]. This highlights the crucial role of geometric confinement in the balance between magnetic and hydrodynamic forces driving the assembly process. In addition to shifting the onset of stability, confinement also affects the spatial organization of the resulting structures, similarly to what was observed for perturbating fields. Specifically, the periodicity of the layered pattern increases significantly with the gap height *h*, following a power‐law dependence with an exponent close to 1, as shown in Figure 6b.

Earlier magnetostatic models by Cutillas et al. [56], which estimate the equilibrium period by minimizing dipolar interaction energy in the absence of flow, predict a weaker dependence (≈ *h* ^0.5^) and fail to capture the influence of shear. In contrast, the chain‐tilting model recently proposed by Morillas et al. [44], which accounts for the inclination of field‐assembled chains under flow, predicts a linear scaling consistent with experimental observations. Despite this agreement in scaling, quantitative discrepancies remain, likely due to model simplifications and the neglect of higher‐order interactions. In this sense, the shear‐induced route differs qualitatively from the field‐induced one, where the final periodicity is governed by a time‐averaged dipolar landscape and exhibits a weaker, sublinear dependence on confinement. A direct comparison of both trends is provided in Note S7.

### 2.3 Programmable Cell Confinement in Layered Hydrogels

**FIGURE 7:**
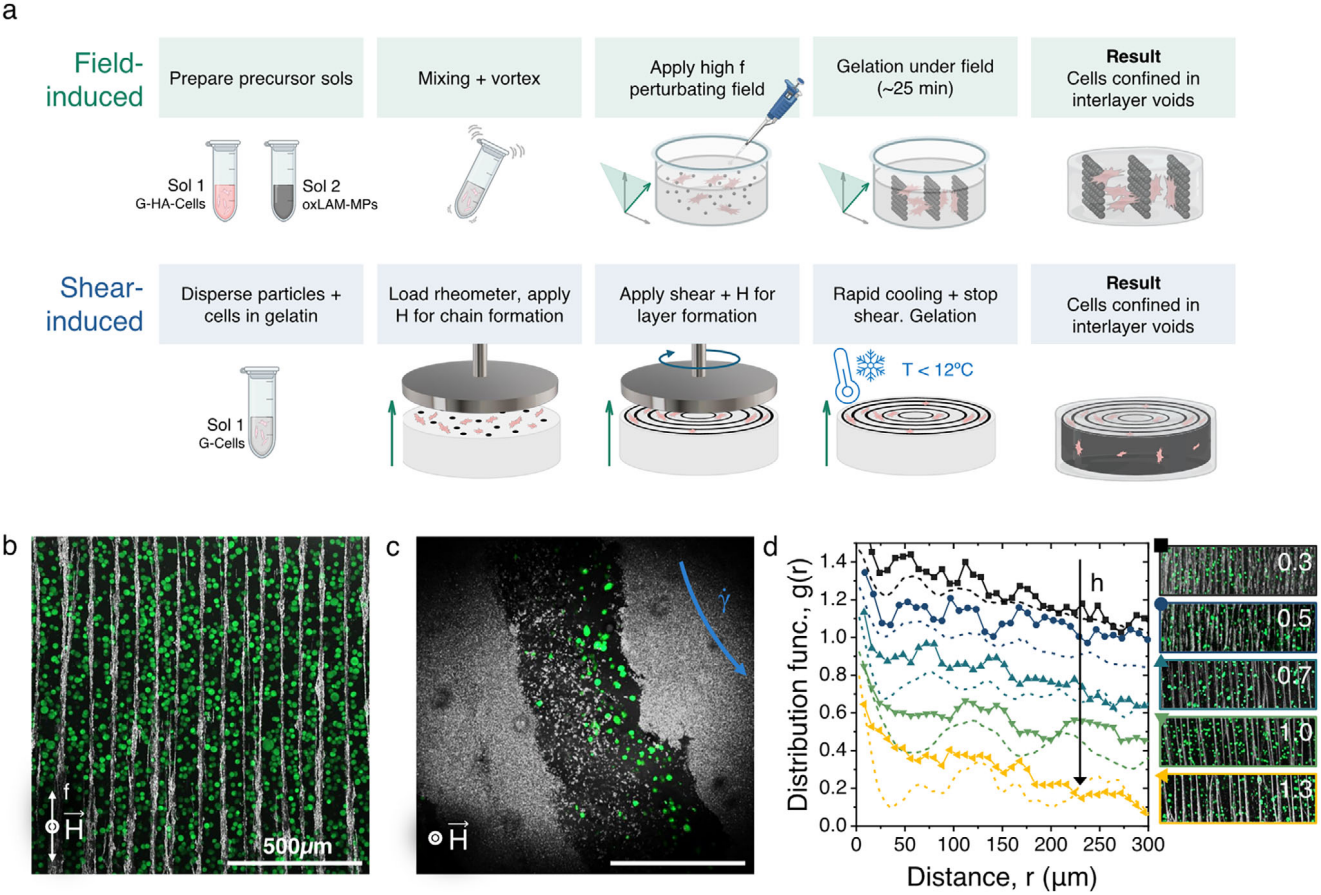
Programmable cell confinement in layered hydrogels. (a) Process diagram of both field‐ and shear‐induced strategies for cell placement in bidirectional anisotropic hydrogels. Bottom row shows confocal micrographs of spatial confinement of HFs cells (green) within particle‐based lamellar structures (white) using: (b) perturbating‐field‐induced patterning and (c) combined shear flow and uniaxial steady field. (d) Distribution profiles of particles (dashed lines) and cells (solid lines and symbols) measured at different sample heights in the range *h*  = 0.3–1.3 mm, illustrating controllable structural periodicity and confinement efficiency. Curves are vertically shifted for easier visualization. Created with BioRender.com.

In this section, we demonstrate that lamellar structures formed by particle‐based assembly can spatially confine cells within well‐defined regions of a hydrogel matrix. Human fibroblasts (HFs) were used as a model cell line to evaluate whether these structures can guide cell positioning in three‐dimensional environments. Two different strategies were explored, corresponding to the two assembly routes described above and schematized in Figure 7a.

In the first strategy, based on perturbating magnetic fields, we employed a biocompatible gelling matrix composed on a formulation of oxidized‐laminarin and gelatin polymers. This hydrogel system undergoes gelation via a bond‐shifting mechanism involving Schiff base formation, enabling the rapid formation of stable structures that preserve the spatial arrangement imposed by the perturbating fields. Static layers are assembled during the sol phase using high Mn_f_ perturbating fields, with this field maintained until the matrix fully gelled (≈25 min, see Experimental section for details). Figure 7b shows a confocal micrograph of the resulting system (layered particle structures shown in white and HFs stained green), where cells are selectively confined between adjacent lamellae. Magnetic layers are sufficiently compact to prevent significant cell overlap with the particle‐rich domains.

In the second strategy, based on the combined action of shear flow and a uniaxial steady field, gelatin was used as the gelling matrix. Gelation in this case was induced by lowering the temperature, promoting physical cross‐linking of the gelatin chains. It is important to note that under this configuration, freezing of the structures must be rapid; otherwise, layers would collapse in the absence of shear. Consequently, the temperature was drastically reduced below 12°C to induce quick gelation while shear was simultaneously removed (see Experimental section and Note S8 for additional details on the rheo‐microscopy and cooling systems). As a result, similar cell confinement was observed in Figure 7c, with cells again accommodating in the interlayer space.

These results establish that both assembly routes can generate particle‐based lamellar scaffolds capable of spatially confining cells. However, for the purposes of the present study, the field‐induced route was selected as the biologically relevant platform, since the shear‐induced configuration requires rapid cooling and rheometer‐based handling conditions that are not well suited for extended cell‐culture studies. Flow‐cytometry measurements under representative structuration conditions showed no appreciable loss of fibroblast viability after exposure to perturbating magnetic fields in the range 1–100 Hz or to shear flow over the typical processing times used here (see Note S9). This analysis was included to isolate the short‐term effect of the external structuration stimuli themselves, rather than to establish the shear‐induced route as a platform for long‐term cell culture.

A major advantage of the field‐induced approach is that the interlamellar spacing can be tuned by controlling the sample height, as described in Section 2.1. To quantify this effect in cell‐laden hydrogels, the periodicity of both the particle and cell distributions was evaluated from confocal stacks by means of a one‐dimensional pair‐correlation analysis along the direction orthogonal to the lamellae. Specifically, we computed the one‐dimensional pair correlation function *g*(*r*) along the axis normal to the layer plane, i.e., the probability of finding a particle at a distance *r* from a reference particle along the selected direction [57]. As shown in Figure 7d, both the particle‐rich bands (dashed lines) and the cell‐populated regions (symbols) display periodicity. These two distributions should be interpreted as complementary: maxima in the particle distribution correspond to minima in cell density, and vice versa. In addition, the cell‐distribution peaks are broader, reflecting the wider interlamellar regions available for cell accommodation relative to the width of the particle‐rich layers.

Importantly, increasing the sample gap leads to a corresponding increase in the periodicity of the cell‐patterned structure, demonstrating that the confinement landscape can be programmed through the geometry of the hydrogel. At the largest interlamellar separations, the periodicity of the cell distribution becomes less pronounced, consistent with weaker geometrical confinement. These results confirm that the proposed strategy enables programmable spatial confinement of cells through control of the internal lamellar architecture, without the need for templates or sequential layer‐by‐layer fabrication.

The introduction of layered particle architectures also modifies the mechanical response of the hydrogel. Comparative rheological and compression measurements performed on bare hydrogels, hydrogels with randomly distributed particles, and hydrogels containing layered structures reveal a clear dependence of the macroscopic mechanical response on the internal organization of the magnetic phase (see Note S10). Thus, the lamellar architecture not only defines cell‐accommodating regions, but also contributes to the overall structure–property response of the material.

**FIGURE 8:**
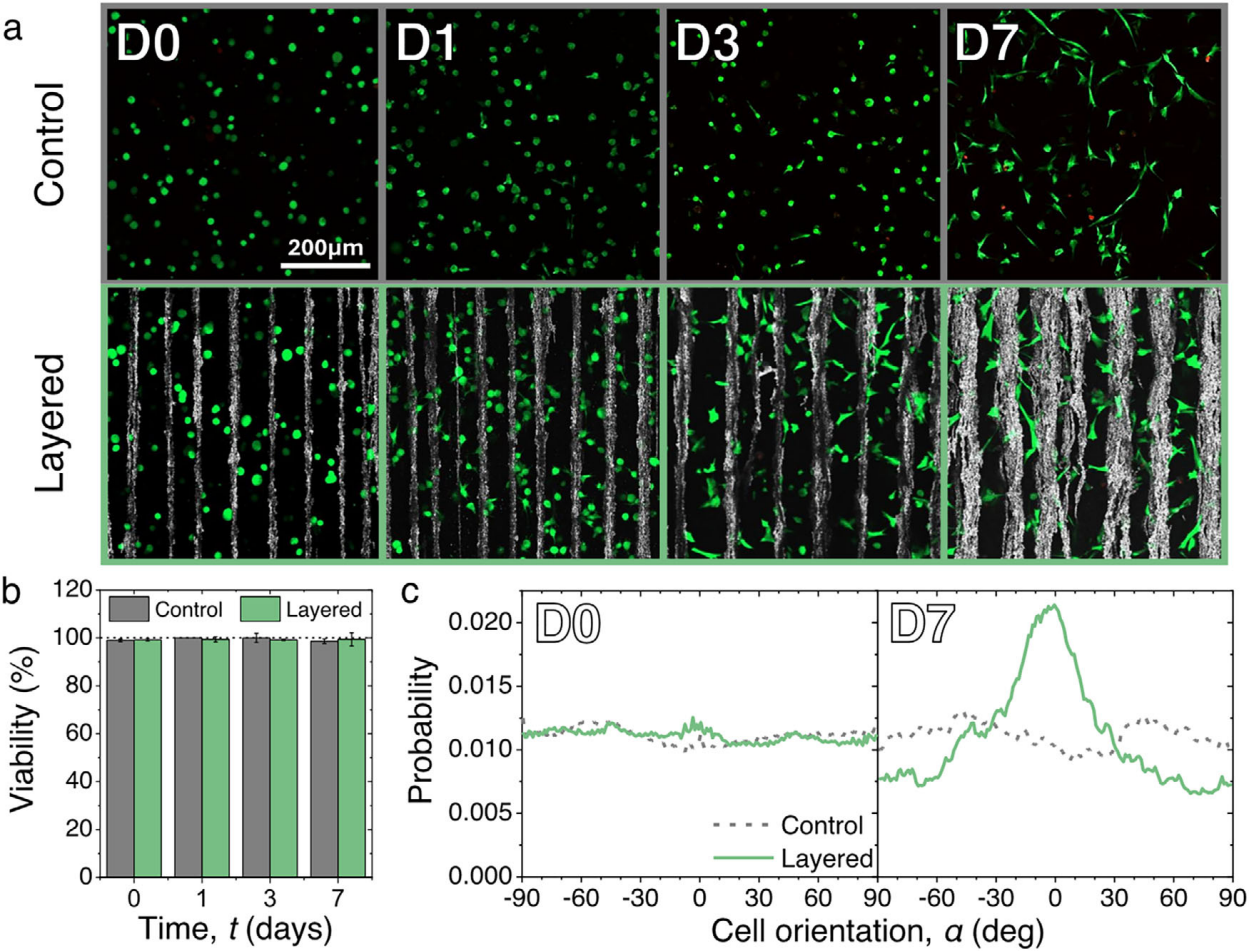
Viability and guided organization of fibroblasts in field‐induced layered hydrogels. (a) confocal images of encapsulated fibroblasts at days 0, 1, 3, and 7 of culture. First row shows a control gel without particles, and second row shows layered samples. Cells are stained green and particles white. (b) Cell viability percentage measured from Live/Dead staining tests for both control and layered samples over time. (c) Cell orientation distribution in the XY‐plane right after structuration (left) and after 7 days (right). Angle is measured from the band direction, meaning 0 degrees parallel to the layer. A strong alignment peak is observed for the layered sample. All results were obtained after averaging at least 3 independent samples.

Long‐term viability in these field‐induced anisotropic gels was also evaluated as a fundamental requirement for tissue‐engineering applications, with the results shown in Figure 8. Human fibroblast cells were encapsulated for 7 days within field‐induced layered hydrogels and in a control sample without particles. Viability after 1, 3, and 7 days was assessed by Live/Dead staining, showing no significant cell death in any case and values above 95% for all configurations (see Figure 8b and Note S11). Long‐term structural stability of the field‐induced layered hydrogels was also monitored under standard culture conditions. As shown in Note S12, the lamellar architecture remained preserved over 7 days, with slight layer bending at long times but no visible particle leakage from the hydrogel matrix.

Even more interestingly, cell morphology analysis reveals a critical aspect of the induced anisotropy. Cell orientation was studied using OrientationJ [58], measuring the angular distribution of the cell channel in confocal microscopy. The results are shown in Figure 8c. Right after structuration, HFs display a rounded shape in all cases, resulting in a nearly isotropic distribution. A similarly isotropic distribution is observed in control samples without particles. By contrast, layered hydrogels reveal a strong alignment peak parallel to the lamellar structure. Thus, the anisotropy imposed by the magnetic scaffold guides cell growth along the established preferred direction.

Taken together, these results highlight the versatility of the layered hydrogels developed here as a platform for programmable cell confinement, guided organization, and architecture‐dependent mechanical response, underscoring their potential for applications in tissue engineering and spatial cell culture systems [59, 60]. A more systematic biological analysis of how interlamellar spacing regulates long‐term cell response remains an important direction for future work.

The proposed magnetic assembly strategies exhibit several technical advantages when compared with other existing methods for generating layered hydrogel architectures. In contrast to light‐based approaches, whose penetration may be limited in dense or optically scattering matrices [61], and electric‐field‐based methods, which can be affected by ionic shielding in physiological media [15], magnetic fields can be applied remotely and penetrate deeply into aqueous systems, making them particularly attractive for volumetric structuring. In addition, the field‐induced route described here does not require photoinitiators or nozzle‐based extrusion and enables the direct formation of interlamellar spaces that can accommodate cells. Under the conditions studied here, this route also shows high fibroblast viability, while cytocompatibility can be further tuned through appropriate particle and matrix selection [62]. These advantages should, however, be balanced against the current limitations of the shear‐induced route, which still requires further development to overcome temperature and sterility constraints. A broader comparison of the technical advantages and limitations of these approaches is provided in Note S13.

## 3 Conclusions

Magnetic‐field‐induced synthesis represents a template‐free and versatile strategy for fabricating anisotropic hydrogels. These magnetically responsive hydrogels not only serve as well‐organized 3D scaffolds that can replicate the complex architecture of natural tissues, but also enable the remote application of magnetomechanical stimuli to modulate cellular behavior under external magnetic fields. As such, they offer both structural anisotropy and dynamic functionality for advanced tissue engineering applications.

While the fabrication of unidirectionally anisotropic hydrogels is relatively well established, the creation of layered hydrogels remains challenging due to the need for sequential magnetic alignment and polymerization steps to achieve complex architectures. Previous efforts have successfully achieved 3D architectures by stacking individual cell sheets, but our study offers a more direct route via structured magnetic suspensions. In this work, we demonstrate two distinct approaches to fabricate lamellar structures using magnetic suspensions as scaffolding materials: i) by applying high‐frequency perturbating fields to quiescent samples, and ii) by superimposing a steady shear flow and a uniaxial steady magnetic field aligned with the velocity gradient direction. In both cases, the magnetic particles undergo directed self‐assembly within a gelling polymer matrix. Upon polymerization, the resulting layered structures are immobilized within the hydrogel network and serve as scaffolds for cell placement while maintaining high fibroblast viability under the conditions studied. Our findings highlight the critical influence of particle concentration and geometric confinement on the layer formation and its periodicity. Furthermore, evaluation of cell morphology over time reveals that confined cells preferentially align parallel to the magnetic lamellae, exhibiting a marked anisotropic organization that is relevant for replicating structurally anisotropic tissues. A systematic analysis of how interlamellar spacing modulates long‐term cell response remains an important next step. The layered architectures not only guide cell positioning, but also produce a distinct mechanical response relative to unstructured controls, further supporting the relevance of internal organization in these materials.

This study focuses specifically on lamellar structures formed from spherical magnetic particles. However, altering particle shape could significantly impact the resulting structures, opening new avenues for anisotropy control. Likewise, the use of triaxial magnetic field configurations—instead of uniaxial and biaxial fields—could enable even more intricate spatial architectures. Finally, while our work centers on the structuring of magnetic particles, future efforts could explore the direct manipulation of magnetically labeled cells to engineer cellular architectures with similar strategies.

## 4 Experimental Section

### 4.1 Magnetic Generation and Imaging System

Setup for magnetic field and flow generation and image acquisition differs for both configurations, given the experimental requirements, as depicted in Figure 2.

Perturbating magnetic fields are generated with a triaxial magnetic field generator, which consists of five coils with mu‐metal yokes that increase field strength. Coils are arranged in pairs along *x* and *y* axes (producing a null gradient in the sample center) and a single coil in *z* to enable sample visualization through microscopy. This configuration allows the generation of magnetic fields along the three directions of space. These coils are coupled to a custom‐made capacitor bank to produce time‐dependent magnetic fields with strengths up to 25 kA m^−1^ and frequencies of 4 kHz. A full description of the field generator and its capabilities can be found in [63]. Perturbating fields are generated superimposing a steady magnetic field in one axis and an orthogonal sinusoidal component. In this setup, micrographs are acquired using a Leica Z6 APO stereomicroscope (18×) in upright configuration coupled to a Photron MiniAX high‐speed camera, for the low magnification experiments, and a Leica DMI3000 B inverted microscope (100×) for the high magnification experiments. The measuring chamber controls the sample height by confining it between two sealed cover glasses.

In the shear‐induced layer formation, experiments were carried out in a rheometer (Anton Paar MCR 501) equipped with a plate‐plate configuration (PP20, 20 mm diameter). The lower plate consisted of a custom‐made 3D‐printed base supporting a transparent glass, which allowed sample visualization through microscopy while the sample was being sheared. Snapshots were captured using a Navitar 1–60123 macroscope (6.5×) mounted on a Photron MiniUX high‐speed camera, with an LED ring providing homogeneous planar illumination. In this configuration, magnetic field was generated parallel to the upper plate shaft using a coil fed by a Tektronix PWS4305 power supply, which can produce field strengths up to 20 kA m^−1^.

### 4.2 Preparation of Magnetic Suspensions

Magnetic suspensions were prepared by dispersing carbonyl iron particles (EW Grade from BASF) in glycerol‐water mixtures to adjust carrier viscosity and density. These particles have an average diameter of *d*  =  3.5 µm, a density of $\rho_{p}=7.86\;g\cdot cm^{-3}$, and a magnetic contrast factor of β  = 0.65. Additional physicochemical characterization of the carbonyl iron particles, including morphology, size distribution, magnetic response, FTIR, and electrophoretic mobility, is provided in the Note S14. Field‐induced experiments were carried out in 55 wt.% glycerol carrier (η_*c*_ =  7 mPa·s and $\rho_{c}=1.14\;g\cdot cm^{-3}$), while shear‐induced experiments were performed with 87 wt% glycerol samples (η_*c*_ =  106 mPa·s and $\rho_{c}=1.23\;g\cdot cm^{-3}$). Specific field configurations for each experiment are detailed in the next subsections and summarized in Table 1.

### 4.3 Field‐Induced Assembly Conditions

Perturbating fields were applied immediately after pipetting the sample into the holder to avoid particle sedimentation. For phase‐diagram experiments, the field strength was fixed at 8 kA m^−1^, while the frequency was adjusted to satisfy the desired values of θ_*p*_ and Mn_f_. This resulted in frequencies ranging from 0.3 Hz, corresponding to the lowest Mn_f_ and highest θ_*p*_, to 1700 Hz, corresponding to the highest Mn_f_ and lowest θ_*p*_.

### 4.4 Shear‐Induced Assembly Conditions

Magnetic particle suspensions were presheared at $\dot{\gamma }=$ 100 s^−1^ for 60 s to remove any previous mechanical history. A steady magnetic field in the range 5.4–16.3 kA m^−1^ was then applied under quiescent conditions for an additional 60 s to allow chain formation. Layer formation and rheological characterization were subsequently performed under the applied magnetic field by varying the shear rate in the interval $\dot{\gamma }=10^{-2}-10^{3}$ s^−1^. Each data point was recorded for at least 10 s to ensure that the sample reached a steady state and that a fully developed patterned structure could be observed [43]. All measurements were repeated at least three times using freshly prepared samples.

### 4.5 Cell Culture

HFs were cultured under standard conditions (37°C, 5% CO_2_) in DMEM supplemented (DMEMsup)—Dulbecco's Modified Eagle Medium (high glucose, with pyruvate; Gibco) supplemented with fetal bovine serum (10%, Gibco), penicillin‐streptomycin (100 UI ml^−1^; Gibco), and L‐glutamine (1 mM; Gibco) —, with media changes every two days. Upon reaching 85%–95% confluency, cultures were passaged via enzymatic detachment using trypsin. For all experiments, cells were seeded onto standard cell culture‐treated flasks.

### 4.6 Fluorescence Staining

For calcein‐based cell distribution staining, HF pellets were gently resuspended in phosphate‐buffered saline (PBS) to obtain a uniform single‐cell suspension. Calcein AM Green (Invitrogen^TM^) was added at a final concentration of 0.5 µL mL^−1^, and cells were incubated for 5 min at 37°C to enable fluorescent signal development. Samples were then centrifuged at 300 xg for 10 min to remove excess dye, the stained cell pellet was resuspended in DMEMsup and immediately combined with the hydrogel precursor prior to gelation. 3D cell distribution within the hydrogel was visualized using a Zeiss LSM 900 Airyscan 2 confocal microscope equipped with a 488 nm argon laser for Calcein excitation, acquiring z‐stacks to capture spatial organization of the labeled cells throughout the scaffold.

### 4.7 Preparation of Field‐Induced Magnetic Hydrogels

Hydrogels structured using perturbating fields were prepared by first making two separate solutions [14]. Gelation was achieved via dynamic covalent imine bond formation between the aldehyde groups of oxidized‐laminarin and the amino groups of gelatin. The oxidized‐laminarin was prepared as previously reported [64]. Briefly, laminarin (0.617 mmol monomer, 5000 Da; Carbosynt) was oxidized with sodium periodate (2.06 mmol) under N_2_ at room temperature for 5 h. This reaction was quenched with ethylene glycol, dialyzed (3.5 kDa cut‐off) and freeze‐dried to obtain a dry white product.

Solution #1 was prepared by mixing oxidized‐laminarin with DMEMsup, using a vortex mixer (VELP Scientifica) for 30 s, and incubating at 37°C for 30 min to ensure complete dissolution. Carbonyl iron particles (EW Grade from BASF) were added to solution #1 immediately after this incubation for a final concentration of 7.9 w/v% (ϕ= 1 vol%), and the mixture was gently resuspended before proceeding to gel casting. To minimize particle aggregation, the sample was vortexed for 30 s, sonicated for 1 min, and vortexed again for 5 s. This final mixing procedure was repeated before each use to ensure the magnetic particles remained well‐dispersed.

Solution #2 was prepared by mixing gelatin (extracted from porcine skin, type A, with a Bloom number equal to 300; Sigma–Aldrich) with DMEMsup, followed by vortexing for 30 s. The mixture was then incubated at 37°C for 30 min to ensure complete dissolution. Subsequently, 5% v/v hyaluronic acid (HyalOne, Fidia Farmaceutici S.p.A.) and 2.4% v/v calcium chloride (100 mg mL^−1^) were added, and the solution was vortexed again for 30 s before being incubated at 37°C for 10 min. Calcein‐AM–labeled HFs were first mixed into solution #2 at 3 × 10^6^ cells mL^−1^ prior to gelification.

Once both precursor solutions were ready, equal volumes (1:1 ratio) of solution #1 and solution #2 were combined to form the final hydrogel. This 1:1 mixing also diluted the initial cell suspension, resulting in a final cell density of 1.5 × 10^6^ cells mL^−1^. After combining both precursor solutions, the precursor mixture was vortexed for 5 min until a viscosity high enough to minimize particle and cell sedimentation was reached [14]. The sample was then placed in the triaxial field generator, and the perturbating field was immediately applied for 25 min. After this time, the matrix was sufficiently gelled to prevent structural collapse. In all cases, a perturbating magnetic field of 12.3 kA m^−1^ and 100 Hz was applied, with perturbation angles ranging from 54.7° to 70°, a range within which layer formation is obtained under the present conditions (see Table 1).

### 4.8 Preparation of Shear‐Induced Magnetic Hydrogels

Hydrogels structured under combined shear and uniaxial steady magnetic fields were prepared by dispersing carbonyl iron particles (EW Grade from BASF) in gelatin dissolved at 7% w/v in PBS (pH 7.5) for 1 h at 37°C. Once fully solubilized, magnetic particles were added at 10% v/v, followed by Calcein‐AM‐labeled HFs to a final density of 1.5 × 10^6^ cells mL^−1^. A volume of 600 µL of this suspension was loaded into a home‐made confocal rheomicroscope equipped with a parallel‐plate geometry (20 mm diameter), allowed to equilibrate for 60 s under a uniaxial steady magnetic field of 10 kA m^−1^, and was then subjected to a shear rate of 200 s^−1^ while maintaining the field. This corresponds to Mn_s_ =  3.3, which is sufficient to induce layer formation for the gap employed (*h* = 600 µm). Gelation was induced by rapid, uniform cooling using liquid nitrogen delivered through a controlled‐flow dispenser (Note S8), which maintained an even temperature drop across the sample. Upon initiation of cooling, shear was stopped and the sample was allowed to solidify during the rapid temperature drop until gelation was complete.

## Conflicts of Interest

The authors declare no conflicts of interest.

## Supporting information




**Supporting File 1**: adma73847‐sup‐0001‐SuppMat.docx.

**Supporting File 2**: adma73847‐sup‐0002‐MovieS1.mp4.

**Supporting File 3**: adma73847‐sup‐0003‐MovieS2.mp4.

## Data Availability

The data that support the findings of this study are available from the corresponding author upon reasonable request.
