# Supplementary material for: Engineering Layered Magnetic Hydrogels for Cell Placement via Shear and Magnetic Field‐Induced Assembly
Source: Adv Mater. 2026 Jul 1;38(44):e73847. doi: 10.1002/adma.73847 (PMC13449110; doi:10.1002/adma.73847)
Supplement: Supplementary file 1 [file ADMA-38-e73847-s002.docx]

**Supplementary information for**

**Engineering Layered Magnetic Hydrogels for Cell Placement**

**via Shear and Magnetic Field-Induced Assembly**

Guillermo Camacho*, Jose R. Morillas, Jesús García-Gutiérrez, Stefania Nardecchia, Óscar Martínez-Cano, Juan de Vicente*

F2N2Lab, Magnetic Soft Matter Group, Department of Applied Physics, Faculty of Sciences, University of Granada, C/Fuentenueva s/n, 18071 - Granada, Spain

Correspondence to: [guillermocamacho@ugr.es](mailto:guillermocamacho@ugr.es); jvicente@ugr.es

This PDF contains:

**Supplementary Notes**

Supplementary Note 1 (Note S1). Potential energy of interaction under perturbating magnetic fields.

Supplementary Note 2 (Note S2). Particle level simulations.

Supplementary Note 3 (Note S3). Influence of particle loading in the self-assembly under perturbating magnetic fields.

Supplementary Note 4 (Note S4). Aligned chains modelling.

Supplementary Note 5 (Note S5). Magnetostatic calculations of layered structures under perturbating magnetic fields: energy minimization

Supplementary Note 6 (Note S6). Interaction potential between sheared chains.

Supplementary Note 7 (Note S7). Direct comparison of confinement-dependent periodicity in field- and shear-induced layered structures.

Supplementary Note 8 (Note S8). Rapid cooling setup for confocal rheomicroscopy of gelatin hydrogels.

Supplementary Note 9 (Note S9). Cell viability under structuration conditions: flow cytometry.

Supplementary Note 10 (Note S10). Rheological and mechanical characterization of laminarin–gelatin hydrogels.

Supplementary Note 11 (Note S11). Cell viability after encapsulation: Live/Dead assays.

Supplementary Note 12 (Note S12). Layer long-term stability of layered hydrogels.

Supplementary Note 13 (Note S13). Comparison with other layered hydrogel fabrication strategies.

Supplementary Note 14 (Note S14). Physicochemical characterization of carbonyl iron particles.

**Supplementary Movies**

Supplementary Movie 1 (Movie S1). Dynamic oscillations at low $\text{Mn}_{\text{f}}$.

Supplementary Movie 2 (Movie S2). Liquid layers.

**Supplementary References**

**Note S1. Potential energy of interaction under perturbating magnetic fields**

A perturbating magnetic field can be expressed as the superposition of a uniaxial steady field and an orthogonal oscillatory sinusoidal component with amplitudes $H_{st}$ and $H_{os}$:

$$\vec{H}\left( t \right)=H_{st}\hat{z}+H_{os}\sin\omega t\hat{x} ,$$

where, for convenience, the field is assumed to lie in the $XZ$ plane, although the derivation can be adapted to any reference frame.

As a first approximation, we consider only the dipolar term of the interaction and neglect mutual magnetization (i.e., the local field). In this case, the magnetic moment of a sphere of diameter $d$ under the field $\vec{H}(t)$ is:

$$\vec{m}=\frac{\pi d^{3}\beta}{2}\vec{H}(t) ,$$

where $\beta=\frac{\mu_{p}-\mu_{0}}{\mu_{p}+2\mu_{0}}$ is the magnetic contrast factor and $\mu_{p}$ is the permeability of the magnetic particles. The interaction energy of two identical dipoles $\vec{m}$ separated by a distance $r$ is:

$$U^{dip}\left( \vec{r} \right)=\frac{\mu_{0}}{4\pi r^{3}}\left[ \vec{m}\cdot\vec{m}-3\left( \vec{m}\cdot\hat{r} \right)^{2} \right] ,$$

where the terms in brackets can be rewritten in spherical coordinates ($x=r\sin\theta\cos\varphi$, $y=r\sin\theta\sin\varphi$, $z=r\cos\theta$) as:

$$\vec{m}\cdot\vec{m}=m^{2}=\left( \frac{\pi d^{3}\beta}{2} \right)^{2}(H_{st}^{2}+H_{os}^{2}\sin^{2} \omega t)$$

$$\left( \vec{m}\cdot\hat{r} \right)^{2}=\left( \frac{\pi d^{3}\beta}{2} \right)^{2}(H_{st}^{2}\cos^{2} \theta+H_{os}^{2}\sin^{2} \theta\cos^{2} \varphi\sin^{2} \omega t+2H_{st}H_{os}\cos\theta\sin\theta\cos\varphi\sin\omega t)$$

In the high $\text{M}\text{n}_{\text{f}}$ regime, particles cannot follow rapid changes in magnetic field vector due to viscous drag, and therefore experience time-averaged interactions. Averaging the above expressions over time yields:

$$\left\langle\vec{m}\cdot\vec{m} \right\rangle=\left( \frac{\pi d^{3}\beta}{2} \right)^{2}(H_{st}^{2}+{H_{os}^{2}}/2)$$

$$\left\langle\left( \vec{m}\cdot\hat{r} \right)^{2} \right\rangle=\left( \frac{\pi d^{3}\beta}{2} \right)^{2}\left( H_{st}^{2}\cos^{2} \theta+\frac{1}{2}H_{os}^{2}\sin^{2} \theta\cos^{2} \varphi\right).$$

Using trigonometric identities, the time-averaged interaction becomes:

$$\left\langle U^{dip}(\vec{r}) \right\rangle=\frac{\mu_{0}\pi d^{6}\beta^{2}}{16 r^{3}}\left[ H_{st}^{2}\left( 1-3\cos^{2} \theta\right)+\frac{H_{os}^{2}}{2}\left( 1-3\sin^{2} \theta\cos^{2} \varphi\right) \right],$$

Or, in terms of the perturbation angle $\tan\theta_{p}={H_{os}}/{H_{st}}$:

$$\left\langle U^{dip}(\vec{r}) \right\rangle=\frac{\mu_{0}\pi d^{6}\beta^{2}H_{st}^{2}}{16 r^{3}}\left[ 1-3\cos^{2} \theta+\frac{\tan^{2} \theta_{p}}{2}\left( 1-3\sin^{2} \theta\cos^{2} \varphi\right) \right]$$

This interaction energy can become fully attractive in-plane for certain perturbation angles, as discussed in the main text. Fig. S1.1 shows the interaction potential for various $\theta_{p}$ values.

Note that the time-averaged interaction energy can be computed as the sum of each rms component for a linear magnetic material provided cross products of the field vanish ($\left\langle H_{i}H_{j} \right\rangle=\delta_{ij}H_{j}^{2}$) [1]:

$$\left\langle U^{dip}(\vec{H}\left( t \right)) \right\rangle=\left\langle U^{dip}(H_{x}\left( t \right)\hat{x}+H_{y}\left( t \right)\hat{y}+H_{z}(t)\hat{z}) \right\rangle=U^{st}\left( \sqrt{\left\langle H_{x}^{2} \right\rangle}\hat{x} \right)+U^{st}\left( \sqrt{\left\langle H_{y}^{2} \right\rangle}\hat{y} \right)+U^{st}\left( \sqrt{\left\langle H_{z}^{2} \right\rangle}\hat{z} \right)$$

with $U^{st}(H)$ the interaction energy under a steady field $H$ (Eq. 1 in the main text).

**Figure S1.1.- Angular dependence of the time-averaged dipolar interaction under perturbating magnetic fields.** Spherical plots of the interaction energy between two dipoles under high-frequency perturbating magnetic fields with different perturbation angles. The sphere represents a constant-distance surface relative to a central particle; green regions indicate attraction and yellow regions indicate repulsion. From left to right: $\theta_{p}=$ 0º (uniaxial steady field), 35º, 55º and 70º.

For the specific case of $\theta_{p}\approx$ 54.7º, the interaction becomes fully isotropic in the field plane, equivalent to that produced by a rotating field [2]. In this isotropic case, the interaction potential can be experimentally determined via the inverted Boltzmann relation applied to the pair-distance histogram $n(r)$ [3]

$$\frac{U\left( r \right)-U\left( r_{ref} \right)}{k_{B}T}=\ln\left[ \frac{n\left( r_{ref} \right)}{n\left( r \right)} \right] ,$$

Results for three different time-averaged field strengths are presented in Fig. S1.2. These experiments were performed in 2D suspensions, with particle pairs deposited on glass under an in-plane magnetic field. We used $1$ µm particles (Dynabeads MyOne™ Carboxilic Acid, ThermoFischer), smaller than in the other experiments reported here (3.5 µm carbonyl iron powder, BASF), as Brownian motion is necessary to adequately sample the interparticle distance distribution. Solid lines in Fig. S1.2 represent the theoretical interaction for $\theta_{p}=$ 54.7º combined with a Hertzian repulsion, whose strength was treated as a fitting parameter.

**Figure S1.2.- Experimental pair interaction potential at the isotropic perturbation angle.** Pair interaction potential for an isotropic perturbating field ($\theta_{p}=$ 54.7º) at three different field strengths. Higher field strengths result in stronger binding between particles, limiting the accessible interparticle distances.

**Note S2. Particle level simulations**

Langevin Dynamics simulations were performed for both layer-assembly configurations: perturbating fields and steady shear combined with a steady field. These simulations reproduced all experimentally observed structures by appropriately setting the magnetic field vector and carrier fluid flow conditions.

The equation of motion was integrated using the Velocity-Verlet algorithm with periodic boundary conditions in the lateral directions and solid walls at the top and bottom of the simulation box [4]. The governing equation is:

$$M_{p}\vec{a}_{i}=-\zeta(\vec{v}_{i}-\vec{u}_{\infty})+\sqrt{2\zeta k_{B}T} \vec{W}\left( t \right)+\sum_{j} \left( \vec{F}_{ij}^{mag}+\vec{F}_{ij}^{rep}+\vec{F}_{ij}^{fric} \right)$$

The left-hand side of the equation represents the inertial force acting on particle $i$ of mass $M_{p}$. The right-hand side includes four main contributions: (i) viscous drag, represented by $-\zeta\vec{v}_{i}$, where $\zeta$ is the Stokes friction coefficient, (ii) carrier flow drag $\zeta\vec{u}_{\infty}$, with a linear flow profile $\vec{u}_{\infty}=\dot{\gamma}z_{i}\hat{x}$ when a shear rate $\dot{\gamma}$ is imposed, (iii) thermal fluctuations, modeled as a stochastic force $\sqrt{2\zeta k_{B}T} \vec{W}\left( t \right)$, where $\vec{W}(t)$ is a Gaussian white noise term with zero mean $\langle W\left( t \right)\rangle=0$ and delta-correlated in time $\langle W\left( t \right)W\left( t' \right)\rangle=\delta\left( t-t' \right)$; and (iv) interparticle interaction forces, including magnetic dipole-dipole interactions dependent on the local magnetic field, solved using the Ewald summation method, a short-range Hertzian repulsion to avoid unphysical particle overlap, and contact friction both between particles and at particle–wall interfaces, following Ruiz-Lopez *et al.* [5]. The custom simulation code uses particle diameter $d$, particle density $\rho_{p}$, and the magnetic force scale $F_{0}=3\mu_{0}\pi d^{2}\beta^{2}H^{2}\text{/}16$ as the basis for nondimensionalization. All simulations reported in this work were run for at least $2\times{10}^{7}$ timesteps with $N=$ 4000 particles.

Simulations in Fig. 3 (main text) were performed in 2D by confining particles to the $XY$ plane, with a magnetic field $\vec{H}\left( t \right)=H_{y}\hat{y}+H_{x}\sin\omega t\hat{x}$. The clustering parameters $S(t)$ and $N_{c}(t)$ were calculated via cluster segmentation, considering particles within 1.05$d$ of each other as belonging to the same cluster. Simulations in Fig. 4 (main text) were carried out in 3D boxes with $\vec{H}\left( t \right)=H_{z}\hat{z}+H_{x}\sin\omega t\hat{x}$. Confinement along the $z$-axis was controlled via the box height. No external flow was imposed in the field-induced configuration ($\vec{u}_{\infty}=0$).

For the layer-assembly configuration based on steady shear and steady field, particle chains were first preassembled under quiescent carrier-fluid conditions with a steady field along the $z$-axis. A shear flow ($\vec{u}_{\infty}=\dot{\gamma}z_{i}\hat{x}$) was then imposed after $2\times{10}^{6}$ timesteps.

It is important to note that the Newtonian assumption applies only to the carrier fluid, consistent with the glycerol–water mixtures used experimentally, and not to the suspension as a whole. The total rheological response of the suspension arises from the combination of the linear viscous contribution of the carrier and the particle stress contribution, which is computed explicitly from the evolving microstructure and interparticle interactions at each simulation step [6]. In this framework, non-Newtonian effects such as shear thinning emerge naturally from the deformation, breakup, and reorganization of field-assembled structures under flow. Therefore, the use of a Newtonian carrier fluid does not imply that shear thinning is neglected at the suspension level; rather, it means that the dissipation of the continuous phase is treated linearly, while the non-Newtonian response of the suspension is captured through the particle dynamics.

**Note S3. Influence of particle loading in the self-assembly under perturbating magnetic fields**

Fig. S3.1 presents the weighted average cluster size $S(t)$ and the number of clusters $N_{c}(t)$ for three different surface fractions—$\phi_{2D}=$0.03, 0.06 and 0.1—collapsed under the aggregation timescale $t_{a}$. Both experiments and simulations show negligible dependence on $\phi_{2D}$ within the range of perturbation angles investigated. Table S3.1 summarizes the scaling exponents obtained for each surface fraction, revealing minimal variation among samples.

**Figure S3.1.- Effect of particle loading on clustering dynamics under perturbating magnetic fields.** Evolution of cluster size $S(t)$ (a) and number of clusters $N_{c}(t)$ (b) for a range of particle surface fractions $\phi_{2D}$ and perturbation angles $\theta_{p}$ in experiments. Panels (c-d) show the corresponding simulation results. Data are scaled by the magnetic aggregation timescale $t_{a}$ and normalized to the initial values of $S$ and $N_{c}$. Panel (e) shows micrographs of assembled structures after 60 s for different $\phi_{2D}$ and $\theta_{p}$.

|  | **Experiments** | | | **Simulations** | | |
| --- | --- | --- | --- | --- | --- | --- |
| $\boldsymbol{\phi}_{\boldsymbol{2}\boldsymbol{D}}$ | $\boldsymbol{z}$ | $\boldsymbol{z'}$ | $\boldsymbol{\Delta}$ | $\boldsymbol{z}$ | $\boldsymbol{z'}$ | $\boldsymbol{\Delta}$ |
| **0.03** | 0.7 | 0.7 | 1 | 0.65 | 0.65 | 1.5 |
| **0.06** | 0.65 | 0.7 | 1.5 | 0.65 | 0.65 | 1.5 |
| **0.1** | 0.7 | 0.7 | 1 | 0.65 | 0.65 | 1.5 |

**Table S3.1.- Scaling exponents of clustering dynamics at different particle surface fractions.** Average scaling exponents for experimental and simulated systems at different particle surface fractions $\phi_{2D}$, extracted from the power-law regime of the clustering parameters. The scaling relations correspond to $S\sim t^{z}$, $N_{c}\sim t^{-z^{'}}$, and $s^{2}n_{s}\sim\left( s/S \right)^{\Delta}$.

**Note S4. Aligned chains modelling**

At low-frequency perturbating fields, thick columns of particles arrange into aligned arrays in the field plane without fully merging. To understand this behavior, we model these particle columns as highly prolate ellipsoids and compute the interaction energy between two such bodies. In the low-frequency regime, the columns can follow the field, making it reasonable to assume that the field vector is always aligned with the long axis of the ellipsoid.

Under this assumption, the magnetic field generated by a uniformly magnetized ellipsoid is given by $\vec{H}_{ell}=-\nabla V$, with:

$$V=-V_{0}\frac{3m}{8\pi H}\int_{\xi}^{\infty} \frac{ds}{\left( s+a^{2} \right)\sqrt{\left( s+a^{2} \right)\left( s+b^{2} \right)^{2}}} ,$$

expressed in ellipsoidal coordinates ($\xi$, $\eta$, $\zeta$) [7,8]. The ellipsoid has a long semi-axis $a$ and short semi-axis length $b$, $m$ is the magnetic moment, $H$ is the external magnetic field, and $V_{0}$ is the potential of the external field defined as $V_{0}=-H z'$, where $z'$ is the coordinate along the arbitrary field axis.

Once $\vec{H}_{ell}$ is obtained, the pair interaction can be estimated by approximating the second ellipsoid, located at $\vec{r}'$, as an identical point dipole $\vec{m}$ aligned with the external field. The interaction energy is then:

$U_{ac}=-\mu_{0}\vec{m}\cdot\vec{H}_{ell}(\vec{r}')$.

In reduced units:

$$u_{ac}=\frac{8\pi}{3m^{2}}U_{ac}=\nabla_{z'} \left( z'\int_{\xi}^{\infty} \frac{ds}{\left( s+a^{2} \right)\sqrt{\left( s+a^{2} \right)\left( s+b^{2} \right)^{2}}} \right)$$

The conversion from ellipsoidal to Cartesian coordinates is given by [9]:

$$\frac{{x'}^{2}}{a^{2}+\xi}+\frac{{y'}^{2}}{b^{2}+\xi}+\frac{{z'}^{2}}{b^{2}+\xi}=1 ,$$

which is solved numerically with $\xi>-b^{2}$.

The modelled configuration is sketched in Fig. S4.1a: two ellipsoids tilted at an angle $\theta$, separated by an in-plane distance $r_{ip}$, and shifted by $r_{lat}$ in the orthogonal direction. Two representative interaction energy maps are shown in Fig. S4.1b,c. For $\theta=$ 0º (Fig. S4.1b), the interaction is isotropically repulsive in the field plane, similar to the case of steady fields, and chains are expected to be randomly distributed in the $XY$ plane. At higher tilting angles (e.g., $\theta=$ 50º, Fig. S4.1c), a long-range attractive region appears in the field plane while short-range interactions remain repulsive. Orthogonal to the field plane, the interaction remains repulsive at all ranges. This explains why aligned in-plane chains form without fully merging, as short-range repulsion prevents coalescence.

The critical tilting angle $\theta_{crit}$ for a given inter-ellipsoid distance $r$ is obtained by analysing $u_{ac}$ as a function of $\theta$ (Fig. S4.1d). This reveals a transition from repulsive to attractive behaviour above $\theta_{crit}$. The complete map of $\theta_{crit}$ as a function of $r_{ip}$ and $r_{lat}$ is shown in Fig. S4.1e. At short distances, no attractive region exists, as $\theta_{crit}$ increases with decreasing $r_{ip}$ or increasing $r_{lat}$.

**Figure S4.1.- Interaction energy landscape for tilted ellipsoids in the aligned-chains model.** a) Schematics of the modeled system; two magnetizable ellipsoids aligned with the instantaneous field, tilted an angle $\theta$. b) Interaction map for $\theta=$ 0º as a function of in-plane ($r_{ip}$) and orthogonal ($r_{lat}$) distances, showing isotropic repulsion. Potential is normalized to the distance decay for easier visualization ($r^{3} u_{ac}$). c) Interaction map for $\theta=$ 50º, showing a long-range attractive region in the field plane and short-range repulsion orthogonally. The boundary between the two regimes is marked by a dotted line. d) Interaction energy vs. tilting angle for different $r_{ip}$; horizontal line indicates the repulsive-attractive transition, and vertical lines mark $\theta_{crit}$ for each curve. e) Critical angle map as a function of $r_{ip}$ and $r_{lat}$.

Modelling the second ellipsoid as a point dipole may be inaccurate at short distances, where chain zippering can occur [10]. Therefore, alternative approaches were tested: treating the second ellipsoid as a uniformly magnetized continuous volume [11] or representing chains as arrays of individual particles in Langevin Dynamics simulations. All approaches yielded qualitatively similar results, with only minor quantitative differences in $u_{ac}$ or $\theta_{crit}$.

**Note S5. Magnetostatic calculations of layered structures under perturbating magnetic fields: energy minimization**

In this section, we derive energy minimization calculations for thin particle layers under high frequency perturbating fields. We use a discrete element approach where each particle is considered individually, being its energy sum of two contributions: a discrete one due to neighboring particles closer than a cut-off distance, and a continuum contribution due to particles beyond it.

Our simulated layer system is depicted in Fig. S5.1. The perturbating field is applied in the $XZ$ plane, with the steady component along the $z$-axis. The layer has finite height ($z$-axis) and width ($y$) but is considered infinite along the $x$-axis, which requires the imposition of a cut-off distance $d_{cut}$ along this direction for the discrete contribution. The goal is to calculate the optimum width for a given height by energy minimization. Given that the layer is infinite along $x$-axis, every slice of the layer in the $YZ$ plane experiences the same interaction energy. This significantly reduces computational cost, as we only compute the interaction between the central slice of particles (the *Field particles*) and all other particles in the layer (*Source particles*). Additionally, we neglect mutual magnetization (local field), considering only external-field magnetization of the particles [12].

**Figure S5.1.- Schematic of the layer model used for magnetostatic energy minimization.** A perturbating field is applied in the $XZ$ plane, and the layer extends infinitely along $x$ with finite width $e$ and height $h$. The central slice of the layer (dark red, *Field particles*) interacts discretely with (grey) *Source particles* up to a cut-off distance $d_{cut}$. Beyond this distance, a continuum approximation is used. Vertical and horizontal hatched surfaces represent the Lorentz and Contour contributions, respectively.

The total energy per particle is computed as the sum of discrete and continuum contributions. The discrete term is computed using direct dipolar interactions as derived in Note S1. The continuum contribution is split into two distinct components: (i) Lorentz term ($U_{i}^{L}$), originated by the magnetic charges over the surface left by the cut-off volume (the “Lorentz cavity field”). Because the medium outside this volume is treated as uniformly magnetized, the hole of the discrete region creates a surface charge on its inner wall. (ii) Contour term ($U_{i}^{C}$), caused by the magnetic charges that appear on the external surfaces of the continuous region (demagnetizing field), where the magnetized object meets the vacuum permeability. Thus, the total energy for a field particle will be calculated as:

$$U_{i}=U_{i}^{dip}+U_{i}^{L}+U_{i}^{C}$$

It should be noted here that the Lorentz contribution will only need the $x$-component of the field and the contour contribution will only require the $z$-component, given that the average of the cross products is zero. The calculation of each of these contributions is detailed below.

**Discrete contribution.** This contribution is computed using the interaction derived in Section S1 for each pair of particles.

$$U^{dip}=\frac{\mu_{0}\pi d^{6}\beta^{2}H_{st}^{2}}{16}\frac{1}{2} \sum_{i} \sum_{j\neq i} \frac{1}{r^{3}}\left[ 1-3\frac{r_{z}^{2}}{r^{2}}+\frac{\tan^{2} \theta_{p}}{2}\left( 1-3\frac{r_{x}^{2}}{r^{2}} \right) \right] ,$$

where it has been used that $\cos\theta={r_{z}}/r$ and $\sin\theta\cos\varphi={r_{x}}/r$. The index $i$ sums over the *Field* particles, while $j$ sums over the *Source* particles. Finally, normalizing by the energy scale $U=\frac{\mu_{0}\pi d^{3}\beta^{2}H_{st}^{2}}{16}\bar{U}$, we have:

$$\bar{U}=\frac{1}{2} \sum_{i} \sum_{j\neq i} \frac{d^{3}}{r^{3}}\left[ 1-3\frac{r_{z}^{2}}{r^{2}}+\frac{\tan^{2} \theta_{p}}{2}\left( 1-3\frac{r_{x}^{2}}{r^{2}} \right) \right]$$

This expression is applied to compute the interaction energy between pairs of particles closer than $d_{cut}$. This effectively defines a cut-off volume depicted as filled of particles in Fig. S5.1.

**Continuum contribution.** The *source particles* outside the cut-off volume are treated as a continuum; therefore the interaction energy in this case will be the energy of a dipole (*field particle*) in the magnetic field generated by the continuous source distribution: $-\mu_{0} \vec{m}\cdot\vec{H}_{cont}$. For the calculation of this scalar product, we must account for the fact that cross products do not contribute to the average energy, since $\left\langle H_{i}H_{j} \right\rangle=\delta_{ij}H_{j}^{2}$.

This continuum contribution comes from the source magnetic charges that appear on the outer surface of the layer (contour term) and on the inner surfaces left by the cut-off volume considered in the previous section (Lorentz term). When a body is magnetized, its induced magnetization acts as a source of magnetic field:

$$\vec{H}=\frac{1}{4\pi}\int_{V_{p}} \rho_{M}\frac{\vec{r}}{r^{3}}dV+\frac{1}{4\pi}\int_{S_{p}} \varrho_{M}\frac{\vec{r}}{r^{3}}dS$$

where $\rho_{M}=-\nabla\cdot\vec{M}$ is the magnetic charge density and $\varrho_{M}=\hat{n}\cdot\vec{M}$ the surface magnetic charge density, with $\hat{n}$ the normal vector of the surface pointing outward, and $\vec{M}$ the magnetization of the layer. $V_{p}$ and $S_{p}$ are the volume and surfaces of interest. In this sense, we consider the two continuous contributions mentioned before: the vertical surfaces determined by the cut volume (Lorentz), and the upper and lower caps of the layer (contours). These surfaces are represented with a hatched pattern in Fig. S5.1.

**Lorentz energy.** The uniform magnetization resulting from neglecting the local field implies that $\rho_{M}=0$ and therefore there is no volumetric contribution. We have only a surface contribution due to the $x$-component of the external field. Furthermore, the surface charge is given by $\varrho_{M}=\pm{\phi_{a}M_{x}}/\sqrt{2}$ $\phi_{a}$, where the $\pm$ sign indicates that the surface charges are equal and opposite on opposing surfaces. In this expression, $\phi_{a}$ is the packing volume fraction, which scales the magnetization, and $M_{x}$ the magnetization of a particle due to $H_{os}$ ($M_{x}=3\beta H_{os}$). The factor $1/\sqrt{2}$ is introduced to represent the rms value as it is an oscillatory component.

$$H_{x}=\frac{\varrho_{x}}{4\pi}\int_{0}^{h} \int_{0}^{e} \frac{x dy^{'}dz'}{\left( x^{2}+y^{2}+z^{2} \right)^{3/2}}$$

with $x=x_{i}-x^{'}=\pm d_{cut}$, $y=y_{i}-y'$ and $z=z_{i}-z'$. $e$ and $h$ are the width and height of the layer. Introducing the change of variables:

$$\bar{y}=\frac{y_{i}-y'}{d_{cut}}=\frac{y}{d_{cut}}, d\bar{y}=-\frac{dy^{'}}{d_{cut}}, \bar{z}=\frac{z_{i}-z'}{d_{cut}}=\frac{z}{d_{cut}}, d\bar{z}=-\frac{dz^{'}}{d_{cut}}$$

the limits become:

$$\bar{0}_{e}=\frac{y_{i}}{d_{cut}}, \bar{e}=\frac{y_{i}-e}{d_{cut}}, \bar{0}_{h}=\frac{z_{i}}{d_{cut}}, \bar{h}=\frac{z_{i}-h}{d_{cut}}$$

and the integral to solve is

$$H_{x}=\frac{\varrho_{x} \bar{x}}{4\pi}\int_{\bar{0}_{h}}^{\bar{h}} \int_{\bar{0}_{e}}^{\bar{e}} \frac{d\bar{y} d\bar{z}}{\left( \bar{x}^{2}+\bar{y}^{2}+\bar{z}^{2} \right)^{3/2}}=$$

$$=\frac{\varrho_{x} \bar{x}}{4\pi}\int_{\bar{0}_{h}}^{\bar{h}} \left[ \frac{\bar{e} d\bar{z}}{\left( \bar{x}^{2}+\bar{z}^{2} \right)\sqrt{\bar{x}^{2}+\bar{e}^{2}+\bar{z}^{2}}}-\frac{\bar{0}_{e} d\bar{z}}{\left( \bar{x}^{2}+\bar{z}^{2} \right)\sqrt{\bar{x}^{2}+\bar{0}_{e}^{2}+\bar{z}^{2}}} \right]$$

Integrating again:

$$H_{x}\left( \varrho_{x},y_{i},z_{i} \right)=\frac{\varrho_{x} \bar{x}}{4\pi\left| \bar{x} \right|}\left[ \mathrm{atan} \left( \frac{\bar{e} \bar{h}}{\left| \bar{x} \right|\sqrt{\bar{x}^{2}+\bar{e}^{2}+\bar{h}^{2}}} \right)-\mathrm{atan} \left( \frac{\bar{e} \bar{0}_{h}}{\left| \bar{x} \right|\sqrt{\bar{x}^{2}+\bar{e}^{2}+\bar{0}_{h}^{2}}} \right)-\mathrm{atan} \left( \frac{\bar{0}_{e} \bar{h}}{\left| \bar{x} \right|\sqrt{\bar{x}^{2}+\bar{0}_{e}^{2}+\bar{h}^{2}}} \right)+\mathrm{atan} \left( \frac{\bar{0}_{e} \bar{0}_{h}}{\left| \bar{x} \right|\sqrt{\bar{x}^{2}+\bar{0}_{e}^{2}+\bar{0}_{h}^{2}}} \right) \right]$$

And therefore, the contribution per particle to the total energy is:

$$U_{i}^{L}(\varrho_{x},y_{i},z_{i})=-\mu_{0} m_{x} H_{x}(\varrho_{x},y_{i},z_{i})$$

Considering both surfaces, the total Lorentz energy is:

$$U^{L}=\sum_{i}^{N_{p}} \left[ U_{i}^{L}\left( \frac{\phi_{a}M_{x}}{\sqrt{2}}, y_{i},z_{i} \right)+U_{i}^{L}\left( -\frac{\phi_{a}M_{x}}{\sqrt{2}}, y_{i}, z_{i} \right) \right]$$

Since $\bar{x}=\frac{x_{i}\pm d_{cut}}{d_{cut}}=\pm1$ for all *Field particles* at a distance $\pm d_{cut}$ along $x$-axis (note that $x_{i}=0$), $U_{i}^{L}\left( \phi_{a}M_{x}/\sqrt{2} , y_{i},z_{i} \right)=U_{i}^{L}\left( -\phi_{a}M_{x}/\sqrt{2}, y_{i}, z_{i} \right)$ and the total reduced Lorentz energy is:

$$\bar{U}^{L}=\sum_{i}^{N_{p}} 2 \bar{U}_{i}^{L}\left( \phi_{a}M_{x}/\sqrt{2} , y_{i},z_{i} \right)$$

$$=-\frac{6\phi_{a}}{\pi}\left( \frac{H_{os}}{H_{st}} \right)^{2}\sum_{i}^{N_{p}} \left[ \mathrm{atan} \left( \frac{\bar{e} \bar{h}}{\sqrt{1+\bar{e}^{2}+\bar{h}^{2}}} \right)-\mathrm{atan} \left( \frac{\bar{e} \bar{0}_{h}}{\sqrt{1+\bar{e}^{2}+\bar{0}_{h}^{2}}} \right)-\mathrm{atan} \left( \frac{\bar{0}_{e} \bar{h}}{\sqrt{1+\bar{0}_{e}^{2}+\bar{h}^{2}}} \right)+\mathrm{atan} \left( \frac{\bar{0}_{e} \bar{0}_{h}}{\sqrt{1+\bar{0}_{e}^{2}+\bar{0}_{h}^{2}}} \right) \right]$$

which can be written in terms of the perturbation angle with $\tan\theta_{p}=H_{os}\text{/}H_{st}$.

**Contours energy.** Let us now calculate the contribution of the upper and lower caps. We need to compute the field along the $z$-direction generated by each cap due to its surface magnetic charge density $\varrho_{z}=\pm\phi_{a}M_{z}$, with $M_{z}$ the particle magnetization due to $H_{st}$ ($M_{z}=3\beta H_{st}$). Therefore, we can calculate the generated field $H_{z}$ by integrating over the two segments $[-\infty,-d_{cut}]$ and $\left[ d_{cut},+\infty\right]$ (Top and bottom hatched surfaces in Figure S5.1). We start with the first segment:

$$H_{z}=\frac{\varrho_{z}}{4\pi}\int_{-\infty}^{-d_{cut}} \int_{0}^{e} \frac{z dy^{'}dx'}{\left( x^{2}+y^{2}+z^{2} \right)^{3/2}}$$

Using the same change of variables as before:

$$\bar{x}=\frac{x_{i}-x^{'}}{d_{cut}}=\frac{x}{d_{cut}}, d\bar{x}=-\frac{dx^{'}}{d_{cut}}, \bar{y}=\frac{y_{i}-y^{'}}{d_{cut}}=\frac{y}{d_{cut}},$$

$$d\bar{y}=-\frac{dy^{'}}{d_{cut}}, \bar{z}=\frac{z_{i}-z^{'}}{d_{cut}}=\frac{z}{d_{cut}}, d\bar{z}=-\frac{dz^{'}}{d_{cut}}$$

The integral limits change accordingly:

$$-\bar{\infty}=\frac{x_{i}+\infty}{d_{cut}}=+\infty, {-\bar{d}}_{cut}=\frac{x_{i}+d_{cut}}{d_{cut}}=1, \bar{0}=\frac{y_{i}}{d_{cut}}, \bar{e}=\frac{y_{i}-e}{d_{cut}}$$

Solving the integral analogously to the Lorentz case, we obtain:

$$H_{z}\left( \varrho_{z},y_{i},z_{i} \right)=\frac{\varrho_{z} \bar{z}}{4\pi\left| \bar{z} \right|}\left[ \mathrm{atan} \left( \frac{\bar{e}}{\left| \bar{z} \right|\sqrt{1+\bar{e}^{2}+\bar{z}^{2}}} \right)-\mathrm{atan} \left( \frac{\bar{e}}{\left| \bar{z} \right|} \right)-\mathrm{atan} \left( \frac{\bar{0}}{\left| \bar{z} \right|\sqrt{1+\bar{0}^{2}+\bar{z}^{2}}} \right)+\mathrm{atan} \left( \frac{\bar{0}}{\left| \bar{z} \right|} \right) \right]$$

By symmetry, the contribution of the other top surface [$d_{cut},+\infty$] is identical. However, we must distinguish the values of $\bar{z}$ for the upper and lower caps, $\bar{z}_{u}=\frac{z_{i}-h}{d_{cut}}$ and $\bar{z}_{l}=\frac{z_{i}-0}{d_{cut}}$, respectively. Therefore, the contribution to the total energy per particle is:

$$U^{C}=\sum_{i}^{N_{p}} \left[ U_{i}^{C}\left( \phi_{a}M_{z}, y_{i},\bar{z}_{u} \right)+U_{i}^{C}\left( -\phi_{a}M_{z}, y_{i}, \bar{z}_{l} \right) \right]$$

with

$$U_{i}^{C}\left( \varrho_{z},y_{i},z_{i} \right)=-\frac{\mu_{0}m_{z}\varrho_{z} \bar{z}_{i}}{2\pi\left| \bar{z}_{i} \right|}\left[ \mathrm{atan} \left( \frac{\bar{e}}{\left| \bar{z}_{i} \right|\sqrt{1+\bar{e}^{2}+\bar{z}_{i}^{2}}} \right)-\mathrm{atan} \left( \frac{\bar{e}}{\left| \bar{z}_{i} \right|} \right)-\mathrm{atan} \left( \frac{\bar{0}}{\left| \bar{z}_{i} \right|\sqrt{1+\bar{0}^{2}+\bar{z}_{i}^{2}}} \right)+\mathrm{atan} \left( \frac{\bar{0}}{\left| \bar{z}_{i} \right|} \right) \right] ,$$

In reduced units $U=\frac{\mu_{0}\pi d^{3}\beta^{2}H_{st}^{2}}{16}\bar{U}$, this becomes:

$$\bar{U}^{C}=\frac{12\phi_{a}}{\pi}\sum_{i}^{N_{p}} \left[ \mathrm{atan} \left( \frac{\bar{e}}{\left| \bar{z}_{u} \right|\sqrt{1+\bar{e}^{2}+\bar{z}_{u}^{2}}} \right)-\mathrm{atan} \left( \frac{\bar{e}}{\left| \bar{z}_{u} \right|} \right)-\mathrm{atan} \left( \frac{\bar{0}}{\left| \bar{z}_{u} \right|\sqrt{1+\bar{0}^{2}+\bar{z}_{u}^{2}}} \right)+\mathrm{atan} \left( \frac{\bar{0}}{\left| \bar{z}_{u} \right|} \right)+\mathrm{atan} \left( \frac{\bar{e}}{\left| \bar{z}_{l} \right|\sqrt{1+\bar{e}^{2}+\bar{z}_{l}^{2}}} \right)-\mathrm{atan} \left( \frac{\bar{e}}{\left| \bar{z}_{l} \right|} \right)-\mathrm{atan} \left( \frac{\bar{0}}{\left| \bar{z}_{l} \right|\sqrt{1+\bar{0}^{2}+\bar{z}_{l}^{2}}} \right)+\mathrm{atan} \left( \frac{\bar{0}}{\left| \bar{z}_{l} \right|} \right) \right]$$

Finally, combining the contributions, the total energy per particle is:

$$\bar{U}=\frac{\bar{U}^{dip}+\bar{U}^{L}+\bar{U}^{C}}{N_{p}}$$

**Results.** For the results presented here, a preassembled hexagonal close-packed lattice is assumed, although other lattices were explored with no significant changes in the power-laws discussed next. Maximum packing is considered, $\phi_{a}=$ 0.64, corresponding to the following unit cell dimensions in terms of the particle diameter $d$:

${B_{x}}/d=\sqrt{3}/2$, ${B_{y}}/d=\sqrt{8/3}$, ${B_{z}}/d=1$

The position vector of a particle in this lattice is:

$$\vec{r}\left( l,m,n \right)=\left( l-\frac{2}{3}\text{mod}\left( m,2 \right) \right)B_{x} \hat{x}+m B_{y} \hat{y}+\left( n-\frac{l}{2} \right)B_{z} \hat{z}$$

The goal is to determine the layer width $e$ that minimizes energy for a fixed height. Assuming identical layers, the layer periodicity is $p=e{\phi_{a}}/\phi$, with $\phi$ the total sample volume fraction.

Fig. S5.2 shows the energy curves as a function of layer width for different heights and perturbation angles, using a cut distance $d_{cut}=$ 600 particle diameters. Other values of $d_{cut}$ were tested to ensure independence of results. Taking the energy minimum as the optimal configuration, the width (or periodicity) vs. height curves follow a power law $p\sim h^{\alpha}$ with average exponent $\alpha=$0.4, with no significant dependence on the perturbation angle.

**Fig S5.2.- Energy minimization analysis of layer width and periodicity under perturbating fields.** Top row: Interaction energy per particle $\overline{U}$ vs. layer width $e$ for different heights $h$ and perturbation angles $\theta_{p}=$ 25º, 54.7º and 75º. Bottom row: Optimal layer width $e$ vs. height $h$, obtained from the configuration that minimizes interaction energy. Layer height and width are expressed in terms of number of particles.

**Note S6. Interaction potential between sheared chains**

Upon application of a shear flow, chains initially aligned with the external field in the gradient direction ($z$) tilt up to an angle $\theta$ given by [13]:

$$\tan\theta=\frac{\text{M}\text{n}_{\text{s}}}{8}\left( \frac{L}{d} \right)^{2}$$

where $L$ is the chain length and $d$ is the particle size.

The interaction potential between two chains tilted at an angle $\theta$ was estimated by treating the chains as dipole lines with linear dipole density $m$ (see Fig. S6.1 and Morillas *et al.* [14] for the full derivation), yielding:

$$U\left( \vec{r}' \right)=-\frac{\mu_{0}m^{2}}{4\pi L}\left[ \frac{2x'\sin\theta\cos\theta}{{x'}^{2}+{y'}^{2}}\left( \frac{{Z'}_{+}}{{r'}_{+}}-\frac{2z'}{r'}+\frac{{Z'}_{-}}{{r'}_{-}} \right)+\left( \cos^{2} \theta-\frac{{x'}^{2}\sin^{2} \theta}{{x'}^{2}+{y'}^{2}} \right)\left( \frac{1}{{r'}_{+}}-\frac{2}{r'}+\frac{1}{{r'}_{-}} \right)+\frac{\left( {x'}^{2}-{y'}^{2} \right)\sin^{2} \theta}{\left( {x'}^{2}+{y'}^{2} \right)^{2}}\left( {r'}_{+}-2r'+{r'}_{-} \right) \right],$$

where all distances are scaled by the chain length $L$, with:

${Z'}_{\pm}=z'\pm1$, $r'=\sqrt{{x'}^{2}+{y'}^{2}+{z'}^{2}}$, ${r'}_{\pm}=\sqrt{{x'}^{2}+{y'}^{2}+{Z'}_{\pm}^{2}}$.

Note that $U$ is expressed in the reference frame of the chain, $\vec{r}'$, whereas we usually work in a frame independent of the chain tilting, $\vec{r}$. Therefore, the following variable change must be applied:

$$\hat{x}=\sin\theta\hat{z}^{'}+\cos\theta\hat{x}^{'}, \hat{z}=\cos\theta\hat{z}'-\sin\theta\hat{x}'$$

The resulting interchain force can be determined from the energy expression as:

$$\vec{f}\left( \vec{r}' \right)=-\nabla'U(\vec{r}')$$

Its components are:

$$f_{x'}\left( \vec{r}' \right)=\frac{\mu_{0}m^{2}}{4\pi L^{2}}\left[ \frac{2{x'}^{2}\cos\theta\sin\theta}{{x'}^{2}+{y'}^{2}}\left( -\frac{{Z'}_{+}}{{r'}_{+}^{3}}+\frac{2z'}{{r'}^{3}}-\frac{{Z'}_{-}}{{r'}_{-}^{3}} \right)+2\cos\theta\sin\theta\frac{{y'}^{2}-{x'}^{2}}{\left( {x'}^{2}+{y'}^{2} \right)^{2}}\left( \frac{{Z'}_{+}}{{r'}_{+}}-\frac{2z'}{r'}+\frac{{Z'}_{-}}{{r'}_{-}} \right)+x'\left( \cos^{2} \theta-\frac{{x'}^{2}\sin^{2} \theta}{{x'}^{2}+{y'}^{2}} \right)\left( -\frac{1}{{r'}_{+}^{3}}+\frac{2}{{r'}^{3}}-\frac{1}{{r'}_{-}^{3}} \right)+\frac{\left( {x'}^{2}-3{y'}^{2} \right)x'\sin^{2} \theta}{\left( {x'}^{2}+{y'}^{2} \right)^{2}}\left( \frac{1}{{r'}_{+}}-\frac{2}{r'}+\frac{1}{{r'}_{-}} \right)-\frac{2\left( {x'}^{2}-3{y'}^{2} \right)x'\sin^{2} \theta}{\left( {x'}^{2}+{y'}^{2} \right)^{3}}\left( {r'}_{+}-2r'+{r'}_{-} \right) \right]$$

$$f_{y'}\left( \vec{r}' \right)=\frac{\mu_{0}m^{2}}{4\pi L^{2}}\left[ \frac{2x'y'\cos\theta\sin\theta}{{x'}^{2}+{y'}^{2}}\left( -\frac{{Z'}_{+}}{{r'}_{+}^{3}}+\frac{2z'}{{r'}^{3}}-\frac{{Z'}_{-}}{{r'}_{-}^{3}} \right)-4\cos\theta\sin\theta\frac{x'y'}{\left( {x'}^{2}+{y'}^{2} \right)^{2}}\left( \frac{{Z'}_{+}}{{r'}_{+}}-\frac{2z'}{r'}+\frac{{Z'}_{-}}{{r'}_{-}} \right)+y'\left( \cos^{2} \theta-\frac{{x'}^{2}\sin^{2} \theta}{{x'}^{2}+{y'}^{2}} \right)\left( -\frac{1}{{r'}_{+}^{3}}+\frac{2}{{r'}^{3}}-\frac{1}{{r'}_{-}^{3}} \right)+\frac{\left( 3{x'}^{2}-{y'}^{2} \right)y'\sin^{2} \theta}{\left( {x'}^{2}+{y'}^{2} \right)^{2}}\left( \frac{1}{{r'}_{+}}-\frac{2}{r'}+\frac{1}{{r'}_{-}} \right)-\frac{2\left( 3{x'}^{2}-{y'}^{2} \right)y'\sin^{2} \theta}{\left( {x'}^{2}+{y'}^{2} \right)^{3}}\left( {r'}_{+}-2r'+{r'}_{-} \right) \right]$$

$$f_{z'}\left( \vec{r}' \right)=\frac{\mu_{0}m^{2}}{4\pi L^{2}}\left[ 2x'\cos\theta\sin\theta\left( \frac{1}{{r'}_{+}^{3}}-\frac{2}{{r'}^{3}}+\frac{1}{{r'}_{-}^{3}} \right)+\left( \cos^{2} \theta-\frac{{x'}^{2}\sin^{2} \theta}{{x'}^{2}+{y'}^{2}} \right)\left( -\frac{{Z'}_{+}}{{r'}_{+}^{3}}+\frac{2z'}{{r'}^{3}}-\frac{{Z'}_{-}}{{r'}_{-}^{3}} \right)+\frac{\left( {x'}^{2}-{y'}^{2} \right)\sin^{2} \theta}{\left( {x'}^{2}+{y'}^{2} \right)^{2}}\left( \frac{{Z'}_{+}}{{r'}_{+}}-\frac{2z'}{r'}+\frac{{Z'}_{-}}{{r'}_{-}} \right) \right]$$

The resulting interaction is strongly dependent on the chain tilting angle $\theta$ given by $\text{M}\text{n}_{\text{s}}$. In the absence of shear ($\theta=$ 0), the interaction follows the classical dipolar behavior, where isotropic lateral repulsion dominates. When shear is applied ($\theta>$ 0), an attractive lobe emerges in the flow direction, while the interaction remains repulsive in the perpendicular (vorticity) direction. This mechanism can explain the formation of layers in the flow-field plane, as chains attract along the flow but repel laterally.

**Fig S6.1.-** **Dipole-line model for sheared chains under a magnetic field.** Schematic of a chain under shear and the superposition of a magnetic field, modeled using the dipole line approximation.

**Note S7. Direct comparison of confinement-dependent periodicity in field- and shear-induced layered structures**

To facilitate a direct comparison between the two assembly routes discussed in the main text, Fig. S7.1 compiles in the same representation the dependence of the interlayer periodicity on the sample gap for both field-induced and shear-induced layered structures. Although both methods generate lamellar architectures, the scaling with confinement differs markedly, reflecting their distinct physical origin.

**Fig. S7.1.**- **Direct comparison of interlayer periodicity as a function of sample gap for the two assembly routes.** Experimental interlayer spacing is plotted against confinement gap for field-induced layers and shear-induced layers using the same axes. The field-induced structures exhibit a sublinear increase of periodicity with gap height, whereas the shear-induced structures show an approximately linear dependence. These different trends reflect the distinct interaction mechanisms responsible for layer formation in each case: a time-averaged dipolar attraction under perturbating magnetic fields versus flow-induced tilting and lateral attraction between field-assembled chains under shear. Solid lines are guides to the eye corresponding to the respective power-law fits discussed in the main text.

**Note S8. Rapid cooling setup for confocal rheomicroscopy of gelatin hydrogels**

Gelation of the gelatin hydrogels occurs when the temperature drops below ~ 20ºC due to physical crosslinking of the polymer. A temperature sweep of the matrix, shown in Fig. S8.1a, reveals a rapid and abrupt transition in shear viscosity once a critical threshold is exceeded.

Layers are formed in the sol state under the conditions described in the text, applying a shear rate of $\dot{\gamma}=$ 200 s^-1^ under a magnetic field $H=$ 10 kA·m^-1^ ($\text{M}\text{n}_{\text{s}}=$ 3.3), generated by an external coil mounted on the rheometer (see Fig. S8.1b). To preserve the layered structures after gelation, the temperature must be decreased rapidly to induce a quick transition; otherwise, the structures disintegrate in the absence of shear. The rheometer is coupled to a confocal microscope to observe the magnetic structures and dyed cells, which requires a glass bottom plate for optical access. This setup limits the use of conventional Peltier cells capable of rapid cooling. To overcome this limitation, a custom-made cooler was designed and 3D-printed to store liquid nitrogen. The cooler can be inserted into the coil and features inner ventilation holes that quickly cool the region it surrounds, including the plate and sample, as shown in Fig. S8.1b.

**Fig. S8.1**.**- Rapid cooling setup for preserving shear-induced layers.** a) Temperature sweep of the gelatin hydrogels, performed using an Anton Paar MCR302 rheometer equipped with a Peltier plate for precise temperature control. b) Schematic of the experimental setup for layer formation in gelatin hydrogels. Observation with a confocal microscope limits the use of Peltier plates, which is overcome by employing a custom-made cooler containing liquid nitrogen.

**Note S9. Cell viability under structuration conditions: flow cytometry**

To determine whether the external stimuli used during the structuration step—namely time-dependent magnetic fields and shear flow—could by themselves compromise cell survival over the processing times relevant to this work, short-term cell viability was quantified by flow cytometry. This analysis was specifically designed to isolate the possible effect of the structuration conditions prior to hydrogel encapsulation, since the corresponding protocols require exposing cells for several minutes to magnetic fields or shear flow outside the incubator environment (37 °C and 5% CO_2_).

Flow-cytometry experiments were performed using a BD FACSAria III cytometer. In the first set of experiments, human fibroblasts (HFs) were suspended in 1.5 mL of DMEMsup medium at a density of $5\times{10}^{5}$cells mL^-1^ and exposed for 10 min to perturbating magnetic fields of 1, 10, and 100 Hz at $H=12.3$kA·m^-1^ and $\theta_{p}={70}^{\circ}$. In the second set of experiments, 600 $\mu$L of an equivalent HF suspension were placed in the rheometer and subjected for the same 10 min to a shear rate of $\dot{\gamma}=200$s^-1^, corresponding to a shear stress of $\tau=0.58$ Pa.

After exposure to the magnetic field or shear flow, the cells were stained with 50 $\mu$L of fluorescein diacetate (FDA, Sigma-Aldrich) and propidium iodide (PI, 95%, Acros Organics) and subsequently analyzed by flow cytometry [15]. FDA was used as the live-cell marker, since it reports intracellular esterase activity in viable cells, whereas PI was used as the dead-cell marker because it labels membrane-compromised cells.

The results are shown in Fig. S9.1, together with the corresponding control sample not exposed to any external stimulus. In all cases, the measured viability remained very close to the control value and close to 100%, indicating that the magnetic-field and shear conditions employed during the typical structuration times used in this work do not produce any appreciable loss of viability. In this sense, the impact of these stimuli during the relevant processing window can be considered negligible within the sensitivity of the assay.

These observations are consistent with previous reports showing negligible viability loss in different cell lines exposed to time-dependent magnetic fields of comparable frequency and intensity for substantially longer times [16,17]. Similarly, comparable shear-stress levels have also been reported to preserve high cell viability [18].

Taken together, these results define an experimentally relevant viability window for the magnetic-field frequencies and shear conditions employed in the present study, although they are not intended to constitute an exhaustive screening of all possible operating parameters.

**Fig. S9.1**.- **Flow-cytometry analysis of cell viability under structuration conditions.** a) Forward-scatter-area versus side-scatter-area plot showing the gating strategy used to isolate the fibroblast population from cell debris and aggregates. b) Representative live/dead discrimination plot of the gated population using fluorescein diacetate (FDA) as live-cell marker and propidium iodide (PI) as dead-cell marker. c) Percentage of viable cells after exposure of HF suspensions to perturbating magnetic fields of 1, 10, and 100 Hz, and to shear flow at $\dot{\gamma}=200$s^-1^, for 10 min. Results are compared with a control sample maintained under standard culture conditions (37 °C and 5% CO_2_). In all cases, viability remained close to 100%.

**Note S10. Rheological and mechanical characterization of laminarin–gelatin hydrogels**

To evaluate the mechanical consequences of particle structuring, the biocompatible laminarin–gelatin hydrogels were characterized using an Anton Paar MCR 501 rheometer equipped with the triaxial magnetic field generator and a plate–plate geometry. Three complementary experiments were performed: (i) small-amplitude oscillatory shear (SAOS) immediately after precursor mixing to monitor gelation kinetics ($f=1$ Hz), (ii) frequency-sweep SAOS on fully gelled samples to determine the linear viscoelastic response, and (iii) uniaxial compression tests at constant crosshead velocity ($v=$ 5 µm·s^-1^) to determine the Young’s modulus.

Shear tests were performed within the linear viscoelastic regime ($\gamma_{0}=$ 20%), as confirmed by amplitude sweeps, using a measuring gap of 300 µm. Samples for shear measurements were gelled in situ in the rheometer to minimize handling-induced perturbations. Compression tests were performed on thicker cylindrical samples gelled in molds of 4 mm thickness to avoid finite-thickness artifacts. The Young’s modulus was obtained from the slope of the stress–strain curve in the linear response region.

All tests were carried out on three different hydrogel configurations (three independent replicates for each): (i) hydrogel without magnetic particles (HG), (ii) hydrogel containing randomly distributed magnetic particles (Random MP HG), and (iii) hydrogel containing layered particle structures (Layered MP HG). For the latter configuration, a perturbating magnetic field of $H=$ 12.3 kA·m^-1^ and $\theta_{p}={70}^{\circ}$ was applied from the start of the experiment to induce lamellar structuring during gelation.

The results are shown in Fig. S10.1. In all cases, a rapid increase in the viscoelastic moduli is observed during the first minutes after mixing, which is essential for preserving the particle organization once the external field is removed. The bare and randomly structured hydrogels exhibit very similar rheological behavior, indicating that the mere presence of particles at the concentration used here has little influence on the macroscopic response in the absence of field-induced organization. By contrast, the layered samples display systematically higher moduli, consistent with a mechanically distinct response associated with the lamellar architecture of the magnetic phase. In these samples, the magnetic field could be safely removed after 25 min of structuring without any significant change in the subsequent mechanical evolution, indicating that the gel network had become sufficiently developed to preserve the layered structure.

The frequency sweeps confirm a predominantly elastic response in all cases, with $G^{'}\gg G^{''}$over the explored frequency range. The weak frequency dependence of the moduli is consistent with the formation of a well-developed gel network. Compression tests reveal the same qualitative trend, with the layered samples exhibiting higher Young’s modulus than both isotropic controls, whereas the bare and randomly structured hydrogels remain comparatively similar.

Overall, these results support a clear structure–property relationship in these materials: whereas the mere addition of magnetic particles produces only minor mechanical changes under the conditions studied, their organization into lamellar architectures leads to a clearly differentiated viscoelastic and compressive response.

**Fig. S10.1**.- **Rheological and mechanical response of laminarin–gelatin hydrogels with different particle organization.** Comparison between bare hydrogels (black squares, HG), hydrogels containing randomly distributed magnetic particles (blue circles, Random MP HG), and hydrogels containing layered magnetic structures (green triangles, Layered MP HG). a) Time evolution of the storage modulus $G^{'}$(filled symbols) and loss modulus $G^{''}$(open symbols) during gelation, measured by SAOS at $f=$ 1 Hz. b) Frequency dependence of $G^{'}$and $G^{''}$for fully gelled samples in the linear viscoelastic regime. c) Representative stress–strain curves obtained in uniaxial compression at a constant crosshead velocity of $v=$ 5 µm·s^-1^. Solid lines indicate the linear fits used to extract the Young’s modulus, whose values are included in the inset. Created with BioRender.com.

**Note S11. Cell viability after encapsulation: Live/Dead assays**

To assess the cytocompatibility of the field-induced laminarin–gelatin hydrogel system after cell incorporation, cell viability was monitored over time by Live/Dead assays following encapsulation.

Cell viability was evaluated at days 0, 1, 3, and 7 using independently prepared seeded hydrogel samples that were stained prior to confocal microscopy. Following the protocol described in the Experimental section, each hydrogel sample was washed with PBS (pH 7.5) and incubated for 45 min with a staining solution prepared from the Live/Dead kit in PBS. This solution contained calcein AM for live-cell detection and ethidium homodimer-1 for dead-cell detection. According to the staining conditions used here, calcein AM was employed at 1 $\mu$L·mL^-1^ (green channel; excitation/emission $\sim$494/517 nm), whereas ethidium homodimer-1 was used at 4 $\mu$L·mL^-1^ (red channel; excitation/emission $\sim$528/617 nm). After staining, the samples were washed again with PBS to remove excess dye and imaged by confocal microscopy (LSM 900 Airyscan 2, Zeiss).

The results are shown in Fig. S11.1. High viability was observed throughout the studied period in both control and layered laminarin–gelatin hydrogels, with viability values remaining close to 100% up to 7 days of culture. These results indicate that the field-induced hydrogel system provides a cytocompatible environment after encapsulation. Quantification was performed using at least three independent samples for each time point.

As discussed in the main text, the layered samples additionally promote preferential cell orientation as a consequence of magnetic confinement. The purpose of the present note, however, is specifically to document the time-resolved viability after encapsulation, whereas the associated effects on cell spatial organization are interpreted in the corresponding section of the main manuscript.

**Fig. S11.1**.- **Time-resolved viability of encapsulated fibroblasts in laminarin–gelatin hydrogels.** (a) Representative confocal images acquired at day 0 (immediately after structuration), and after 1, 3, and 7 days of culture. The top row corresponds to control hydrogels without magnetic particles, and the bottom row to layered hydrogels structured under perturbating magnetic fields. Live cells are shown in green, dead cells in red, and magnetic particles in white. (b) Viability percentage obtained from cell counting in at least three independent samples for each time point.

**Note S12. Layer long-term stability of layered hydrogels**

The stability of the layered structures encapsulated in the laminarin–gelatin hydrogels was monitored by confocal microscopy at days 0, 1, 3, and 7 under standard culture conditions (DMEMsup medium at 37 °C and 5% CO₂). Quantitative analysis of the particle distribution function over time was used to assess the preservation of the characteristic layered periodicity and structural integrity. The results are shown in Fig. S12.1.

The layered architecture remains clearly identifiable throughout the studied period. After 7 days, slight bending of some layers is observed, consistent with slow relaxation or degradation of the hydrogel matrix, but the characteristic periodicity of the structure is preserved. This bending is visible in confocal images as curvature in lateral projections and as an apparent broadening of the layers in top-view projections. The same effect is reflected in the particle distribution function as a widening of the characteristic peaks, while the peak spacing remains essentially unchanged.

No visible particle release from the hydrogel matrix was detected during the examined period. Overall, these results indicate that the main structural features of the layered scaffold are maintained over the 7-day culture window relevant to the biological experiments reported in this work.

**Fig. S12.1**.- **Long-term structural stability of layered laminarin–gelatin hydrogels. (**a) Representative confocal projections of the layered particle structures at days 0, 1, 3, and 7 under standard culture conditions (DMEMsup medium, 37 °C, 5% CO₂). Top views are shown in the upper panels and lateral views in the lower panels. The particle signal is shown in white; the fibroblast channel was digitally removed to facilitate visualization of the layered architecture. (b) Particle distribution function at the corresponding time points. Slight layer bending at long times manifests as broader characteristic peaks, while the interpeak spacing remains essentially unchanged, indicating preservation of the characteristic periodicity of the layered scaffold.

**Note S13. Comparison with other layered hydrogel fabrication strategies**

To better contextualize the magnetic assembly routes developed in this work, Table S13.1 compares them with other representative approaches used to generate layered or spatially organized hydrogel architectures, namely lithographic methods, 3D bioprinting, and electro-induced assembly. The comparison focuses on several practically relevant aspects, including the primary actuation mechanism, structural controllability, the mechanism governing layer spacing, biological compatibility, and scalability.

The magnetic routes presented here occupy a distinct niche among these methods. In contrast to light-based approaches, whose penetration may be limited in dense or optically scattering matrices [19], and electric-field-based strategies, which can be affected by ionic shielding in physiological media [20], magnetic fields can be applied remotely and penetrate deeply into aqueous systems, making them attractive for volumetric structuring. In particular, the field-induced route enables template-free formation of lamellar architectures with tunable interlamellar spacing through control of field conditions and geometric confinement, while maintaining high fibroblast viability under the conditions studied here. As with other magnetic hydrogel platforms, cytocompatibility can be further tuned through appropriate particle and matrix selection [21].

At the same time, the comparison also makes explicit the current limitations of the shear-induced route developed here. Although it provides access to a distinct family of layered structures, its present implementation relies on rapid cooling and rheometer-based handling conditions that are not yet optimal for long-term biological use. The purpose of this comparison is therefore not to establish a universally superior method, but rather to clarify the technical scope, strengths, and present limitations of the magnetic assembly strategies introduced in this work.

Overall, this comparison shows that the field-induced magnetic route is especially well suited for template-free volumetric generation of tunable lamellar hydrogels, whereas the shear-induced route expands the accessible structural landscape but still requires further development for biologically sustained use.

| Metric | **Lithography** | **3D Bioprinting** | **Electro-induced assembly** | **Magnetic Field-induced assembly [this work]** | **Shear-induced assembly [this work]** |
| --- | --- | --- | --- | --- | --- |
| **Primary actuating stimulus** | Spatiotemporal UV/Visible light exposure. | Mechanical pneumatic/piston extrusion through a nozzle. | High-voltage electric fields/AC signals. | Unsteady magnetic fields. | Steady magnetic fields coupled with shear flow. |
| **Structural controllability** | Extremely high 2D planar resolution ($<10\mu m$). Any 2D pattern is achievable per layer.  Multiple layers by sequential exposures produce stratified structures susceptible to staircasing and heterogeneities. | Moderate resolution ($\sim100\mu m$). Determined by nozzle diameter and ink yield stress. | High variability:  -Patterning by electrode design (Electrophoresis). Up to $\sim50\mu m$ resolution.  -Polyelectrolyte layered structures due to adsorption and charge reversal.  -Electrically induced self-assembly. | Uniform lamellar structures aligned by the field.  Volumetric control governed by the Mason number (Mn_f_).  Resolution is a few microns (particle size). | Layered structures in the velocity-gradient plane.  Volumetric control governed by the Mason number (Mn_s_). |
| **Mechanism of layer periodicity** | Layer thickness is defined by light exposure (typically 10-500$\mu m$). | Physically defined by the height of the extruded filament (~0.1-1 mm). | Physically defined by electrode geometry.  Chemically defined for polyelectrolytes.  Temporally defined for electrically induced self-assembly. | Spacing and orientation are governed by field conditions and geometric confinement.  Spacing ranges from $\sim20\mu m$ to hundreds of microns.  Very narrow layer width (particle-thick). | Spacing set by geometry.  Spacing ranges from $\sim100\mu m$ to millimeters.  Thicker layers with fluid internal structure. |
| **Biocompatibility** | Many photo-initiators and hydrogels are cell friendly. Toxic photo-initiators must be avoided. Long exposures can generate genetic damage and unreacted monomer leaching. | Generally good for selected materials. High nozzle shear stress can rupture and kill cells. | Potentially cytotoxic nanofillers. Strong fields or Joule heating can harm cells. Exposure must be kept low. | High fibroblast viability demonstrated under the conditions studied. Cytocompatibility can be further tuned through particle and matrix selection. | Not established here for long-term culture conditions; further optimization of matrix chemistry and processing conditions is required. |
| **Scalable production capacity** | Good for 2D/film scale:  Large-area exposure is fast but limited to thin layers.  Building thick samples is slow, bottlenecked by optical attenuation. | Can print cm-scale constructs.  Bottlenecked by printing time: layer-by-layer extrusion. | Typically restricted to thin films.  Limited by Debye electrostatic shielding. | Can form cm-scale gels.  Magnetic field can penetrate deeply. Size determined by field generator size and time determined by gelation time. Easily scalable. | Can form cm-scale gels.  Magnetic field can penetrate deeply. Size determined by flow-field generator size and time determined by gelation time. Easily scalable. |

**Table S13.1**.- **Comparison of representative strategies for the fabrication of layered or spatially organized hydrogel architectures.** The comparison is intended to position the magnetic assembly routes developed in this work relative to other established approaches in terms of actuation mechanism, structural control, spacing definition, biological compatibility, and volumetric fabrication potential.

**Note S14. Physicochemical characterization of carbonyl iron particles**

Carbonyl iron particles used throughout this work were characterized to assess their morphology, size distribution, magnetic response, and field-free dispersion-related properties. Fig. S14.1 compiles SEM micrographs, particle size distribution, FTIR spectra, magnetization curves, and electrophoretic mobility data.

Particle imaging (Fig. S14.1a) was performed using a JEOL JSM-IT800 SHL field emission scanning electron microscope (SEM). Size distribution (Fig. S14.1c) was obtained from microscopy imaging with 724 samples using ImageJ. A polydisperse size distribution with average of $d=$ 3.5 µm is obtained.

Fourier transform infrared spectroscopy (FTIR) was measured using the KBr pellet method with the dry carbonyl iron particle powder in a JASCO FT/IR-6200 spectrometer. Pure iron, the main component of the particles, does not contribute to the FTIR spectrum, so the features observed in Fig. S14.1b are due to impurities, surface effects, or the KBr matrix. The sloping baseline is attributed to Rayleigh or Mie scattering of the IR light by the particles [22]. Although KBr is transparent to IR in the wavenumber range 400-4000 cm^-1^, it is highly hygroscopic, so O-H and H-O-H peaks observed are due to humidity adsorbed on particle surface and KBr. The Fe-O peak around 600 cm^-1^ is originated by surface passivation with a thin layer of oxide in the air [23].

Magnetization curve of the particles was measured using a Quantum Design MPMS XL magnetometer. Results, shown in Fig. S14.1d, reveal an absence of magnetic remanence and an initial susceptibility of $\chi=$ 5.5 ($\mu_{p}=$ 6.5), that yields a magnetic contrast factor of $\beta=\left( \mu_{p}-\mu_{c} \right)\text{/}\left( \mu_{p}+2\mu_{c} \right)=$ 0.65. Magnetic saturation is achieved at $M_{s}=$ 1600 kA·m^-1^ for an external field of $H_{s}=$ 1000 kA·m^-1^.

Electrophoretic mobility measurements, performed using Zetasizer Malvern Nano ZS, reveals an isoelectric point at pH $\approx$ 5.4, rendering a stable solution for the hydrogel precursor.

**Fig. S14.1**.- **Physicochemical characterization of the carbonyl iron particles used in this work.** (a) SEM micrograph. (b) Particle size distribution. (c) FTIR spectrum. (d) Magnetization curve. (e) Electrophoretic mobility as a function of pH. These data are consistent with the use of magnetically soft microparticles and support the absence of significant irreversible aggregation under field-free conditions.

**Supplementary Movies**

**Movie S1. Dynamic oscillations at low** $\text{Mn}_{\text{f}}$**.**

**Filename: S1-Mn0.01_55deg_30fps**

Dynamic oscillations at low $\text{Mn}_{\text{f}}$. In this regime, structures are able to follow the field vector as magnetic interactions dominate over hydrodynamic drag. Movie shows the “aligned chains” phase observed at $\text{M}\text{n}_{\text{f}}=0.01$ and $\theta_{p}={55}^{o}$ with a volume fraction of $\phi=0.1$.

**Movie S2. Liquid layers.**

**Filename: S2-FlowLayerSim**

Particle level simulations of thin, compact and liquid layers formed under shear at $\text{M}\text{n}_{\text{s}}=0.025$ and $25$, respectively. In the first step, samples are structured under a steady field in a quiescent carrier. In the second step, the shear flow is applied.

**Supplementary References**

[1] Martin, J. E., Anderson, R. A. & Williamson, R. L. Generating strange magnetic and dielectric interactions: classical molecules and particle foams. *J. Chem. Phys.* **118**, 1557 (2003).
[2] Halsey, T. C., Anderson, R. A. & Martin, J. E. The rotary electrorheological effect. *Int. J. Mod. Phys. B* **10**, 3019–3027 (1996).
[3] Du, D., Li, D., Thakur, M., & Biswal, S. L. Generating an in situ tunable interaction potential for probing 2-D colloidal phase behavior. *Soft Matter* **9**, 6867 (2013).
[4] Allen, M. P. & Tildesley, D. J. *Computer Simulations of Liquids*. Clarendon (1991).
[5] Ruiz-Lopez, J. A., Prasanna Kumar, S. S., Vazquez-Quesada, A., de Vicente, J. & Ellero, M. Tribological variable-friction coefficient models for the simulation of dense suspensions of rough polydisperse particles. *J. Rheol.* **67**, 541–558 (2023).
[6] Camacho, G., Morillas, J.R. & de Vicente, J. Enhancing magnetorheology through the directed self-assembly under toggled magnetic fields in saturation. *Smart Materials and Structures* **30** (10), 105029 (2021).
[7] Stratton, J. A. *Electromagnetic Theory*. McGraw-Hill (1941).
[8] Nagaoka, Y., Morimoto, Y., & Maekawa, T. Ordered complex structures formed by paramagnetic particles via self-assembly under an ac/dc combined magnetic field. *Langmuir* **27**, 9160-9164 (2011).
[9] Torres-Díaz, I. The ellipsoid-dipole model. Theoretical fundamentals and applications. *Soft Matter* **21**, 6432 (2025).
[10] Faraudo, J., Andreu, J. S., Calero, C., & Camacho, J. Predicting the Self-Assembly of Superparamagnetic Colloids under Magnetic Fields. *Adv. Func. Mat.* **26**, 3837-3858 (2016).
[11] Azari, A., Crassous, J. J., Mihut, A. M., Bialik, E., Schurtenberger, P., Stenhammar, J. & Linse, P. Directed self-assembly of polarizable ellipsoids in an external electric field. *Langmuir* **33**, 13834–13840 (2017).
[12] Sherman, Z. M., Ghosh, D. & Swan, J. W. Field-directed self-assembly of mutually polarizable nanoparticles. *Langmuir* **34**, 7117–7134 (2018).
[13] Martin, J. E. & Anderson, R. A. Chain model of electrorheology. *J. Chem. Phys.* **104**, 4814-4827 (1996).
[14] Morillas, J. R., Martínez-Cano, Ó., Cvek, M., Morris, J. F. & de Vicente, J. Flow-induced layer formation in dipolar non-Brownian suspensions. Submitted.
[15] Ormerod, M. G. Flow Cytometry: A Practical Approach. *OUP Oxford* (2000).
[16] Sun, J., Tong, Y., Jia, Y., Jia, X., Wang, H., Chen, Y., Wu, J., Jin, W., Ma, Z., Cao, K., Li, X., Chen, Z. & Yang, G. Effects of extremely low frequency electromagnetic fields on the tumor cell inhibition and the possible mechanism. *Sci. Rep.* **13**, 6989 (2023).
[17] Pasi, F., Sanna, S., Paolini, A., Alquati, M., Lascialfari, A., Corti, M. E., Di Liberto, R., Cialdai, F., Monici, M. & Nano, R. Effects of extremely low-frequency magnetotherapy on proliferation of human dermal fibroblasts. *Electromagn. Biol. Med.* **35**, 343–352 (2016).
[18] Suwannaphan, T., Kamnerdsook, A., Chalermwisutkul, S., Techaumnat, B., Damrongplasit, N., Traipattanakul, B., Kasetsirikul, S. & Pimpin, A. Effects of shear and extensional stresses on cells: Investigation in a spiral microchannel and contraction−expansion arrays. *ACS Biomater. Sci. Eng.* **11**, 3249–3261 (2025).
[19] Ghazali, H. S., Askari, E., Ghazali, Z. S., Naghib, S. M. & Braschler, T. Lithography-based 3D printed hydrogels: From bioresin designing to biomedical application. *Colloid Interface Sci. Commun.* **50**, 100667 (2022).
[20] Liu, M., Ishida, Y., Ebina, Y., Sasaki, T., Hikima, T., Takata, M. & Aida, T. An anisotropic hydrogel with electrostatic repulsion between cofacially aligned nanosheets. *Nature* **517**, 68–72 (2015).
[21] Pardo, A., Gómez-Florit, M., Barbosa, S., Taboada, P., Domingues, R. M. A. & Gomes, M. E. Magnetic nanocomposite hydrogels for tissue engineering: Design concepts and remote actuation strategies to control cell fate. *ACS Nano* **15**, 175–209 (2021).
[22] Griffiths, P. R. & de Haseth, J. A. Fourier Transform Infrared Spectrometry. *John Wiley & Sons* (2007).
[23] Dong, X., Ma, N., Han, B., Guo, P. & Huang, Y. Improved distribution homogeneity of carbonyl iron particles in magnetorheological elastomers by adding zinc dimethacrylate. *Smart Mater. Struct.* **29**, 025021 (2020).
